# Shotgun Proteomics of Human Dentin with Different Prefractionation Methods

**DOI:** 10.1038/s41598-019-41144-x

**Published:** 2019-03-14

**Authors:** Matthias Widbiller, Helmut Schweikl, Astrid Bruckmann, Andreas Rosendahl, Eduard Hochmuth, Sophia R. Lindner, Wolfgang Buchalla, Kerstin M. Galler

**Affiliations:** 1 Department of Conservative Dentistry and Periodontology, University Hospital Regensburg, Regensburg, Germany; 2 Biochemistry Center Regensburg, Laboratory for RNA Biology, University of Regensburg, Regensburg, Germany; 3 Department of Oral- and Maxillofacial Surgery, University Hospital Regensburg, Regensburg, Germany

## Abstract

Human dentin is not only a composite material of a collagenous matrix and mineral to provide strength and elasticity to teeth, but also a precious reservoir full of bioactive proteins. They are released after demineralization caused by bacterial acids in carious lesions, by decalcifying irrigants or dental materials and they modulate tissue responses in the underlying dental pulp. This work describes a first-time analysis of the proteome of human dentin using a shotgun proteomic approach that combines three different protein fractionation methods. Dentin matrix proteins were extracted by EDTA and separated by sodium dodecyl sulfate polyacrylamide gel electrophoresis (SDS-PAGE), OFFGEL isoelectric focusing (IEF) or strong cation exchange chromatography (SCX). Liquid chromatography tandem mass spectrometry (LC-MS/MS) identified 813 human proteins with high confidence, however, isoelectric focusing turned out to be the most beneficial prefractionation method. All Proteins were categorized based on the PANTHER system and representation analysis revealed 31 classes and subclasses to be overrepresented. The acquired knowledge provides a comprehensive insight into the number of proteins in human dentin as well as their physiological and pathological functions. Thus, the data presented paves the way to the analysis of specific functions of dentin matrix proteins *in vivo* and their potential in tissue engineering approaches to regenerate dental pulp.

## Introduction

Dentin constitutes the major component of teeth and forms a protective shield around the dental pulp. The organic matrix preceding the mineral phase is secreted by odontoblasts during tooth development, where each cell leaves a process behind that becomes embedded in the mineralized tissue, bringing about the tubular architecture of dentin. During dentinogenesis, odontoblasts produce collagen but also non-collagenous signaling molecules, which become fossilized in the matrix and preserve their bioactive potential over a lifetime^1^. Among others, cytokines, growth factors, neurotrophic proteins and extracellular matrix molecules like small integrin-binding ligand N-linked glycoproteins (SIBLINGs), small leucine-rich proteoglycans (SLRPs) and osteocalcin are present in human dentin^1^.

It is known that these signaling molecules are released and allowed to diffuse into the pulpal tissue via the dentinal tubules by decalcification of dentin in carious lesions or by application of alkaline pulp-capping agents or acidic etchants in dentin bonding agents^2,3^. The proteins released from dentin are believed to modulate immunoresponse, to exert chemotactic effects, to stimulate angiogenesis, cell proliferation and differentiation and thus to promote regenerative or reparative processes^4–7^.

Furthermore, dentin matrix proteins are exposed on root canal walls after conditioning with chelating agents such as EDTA. In regenerative endodontic procedures, bioactive proteins might contribute to tissue formation in young patients with immature roots and pulp necrosis after provocation of bleeding into the canal^8,9^. The exact role of bioactive proteins in the complex processes of tissue response remains to be better understood.

The approach of decalcifying the extracellular matrix has also been used to extract proteins from human dentin for laboratory use^6,10^. Several groups applied similar extraction methods to isolate matrix components from dentin powder and determine their composition, however, the efficiency of the applied techniques as well as methodical restrictions regarding protein fractionation limited the protein coverage. So far, not more than 289 proteins have been identified in human dentin^10–12^.

Proteomic analysis of complex samples such as dentin is challenging as protein coverage is highly restricted by ion suppression and a limited loading capacity of the used systems. Thus, a mitigation of sample complexity by separation of proteins into different fractions allows for facilitated analysis by liquid chromatography-tandem mass spectrometry (LC-MS/MS).

A common prefractionation method that has been used for proteomic analysis of human dentin^10–12^ is sodium dodecyl sulfate polyacrylamide gel electrophoresis (SDS-PAGE). With this technique, proteins are denatured by SDS and separated in a polyacrylamide gel according to their molecular weight. The resulting gel lane can be sliced into portions related to the molecular weight, digested trypticly (in-gel) and submitted to mass spectrometry. Another method, OFFGEL isoelectric focusing (IEF), can separate complex protein samples based on their different isoelectric point (pI). With this method, peptides distribute according to their pI in liquid compartments that are connected by a gel with an immobilized pH gradient^13^. Protein fractions can finally be recovered from the liquid phase for further analysis. Strong cation exchange chromatography (SCX) is a third possibility to fractionate complex protein samples at a fixed pH. Peptide mixtures flow through analytical SCX columns with different elution times based on their size and charge. Different fractions can be submitted to LC-MS/MS^14^.

Thus, prefractionation enables a “simplified” mixture of proteins which can be analyzed more successfully by mass spectrometry due to a reduction of signal-to-noise ratio and protein interference. The combination of multiple fractionation methods before tandem mass spectrometry consequently enables a more comprehensive detection and can increase total protein coverage^14^.

Thus, the main focus of this study was to cover the human dentin proteome more comprehensively utilizing different physical and chemical properties of proteins by combination of three prefractionation methods: SDS-PAGE, IEF and SCX. In addition to a comparison of the advanced fractionation methods, proteins detected in human dentin will be organized to identify the most represented protein classes as well as their presumable functions in the dentin pulp complex.

## Results

**Figure 1:**
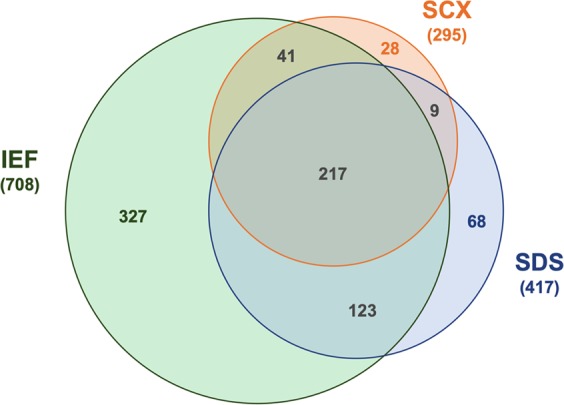
The Venn diagram shows overlapping proteins detected in human dentin by mass spectrometry and combination of different prefractionation methods: isoelectric focusing (IEF), sodium dodecyl sulfate polyacrylamide gel electrophoresis (SDS) and strong cation exchange chromatography (SCX).

Altogether, 813 human proteins were detected in dentin extracts with high confidence (Mascot score ≥30; FDR ≤ 1%). 327 proteins were exclusively discovered after fractionation by isoelectric focusing (40.2%), 68 after gel electrophoresis (8.4%) and 28 after cation exchange chromatography (3.4%). However, 390 proteins (48.0%) were found after two or three prefractionation methods (Fig. 1).

**Figure 2:**
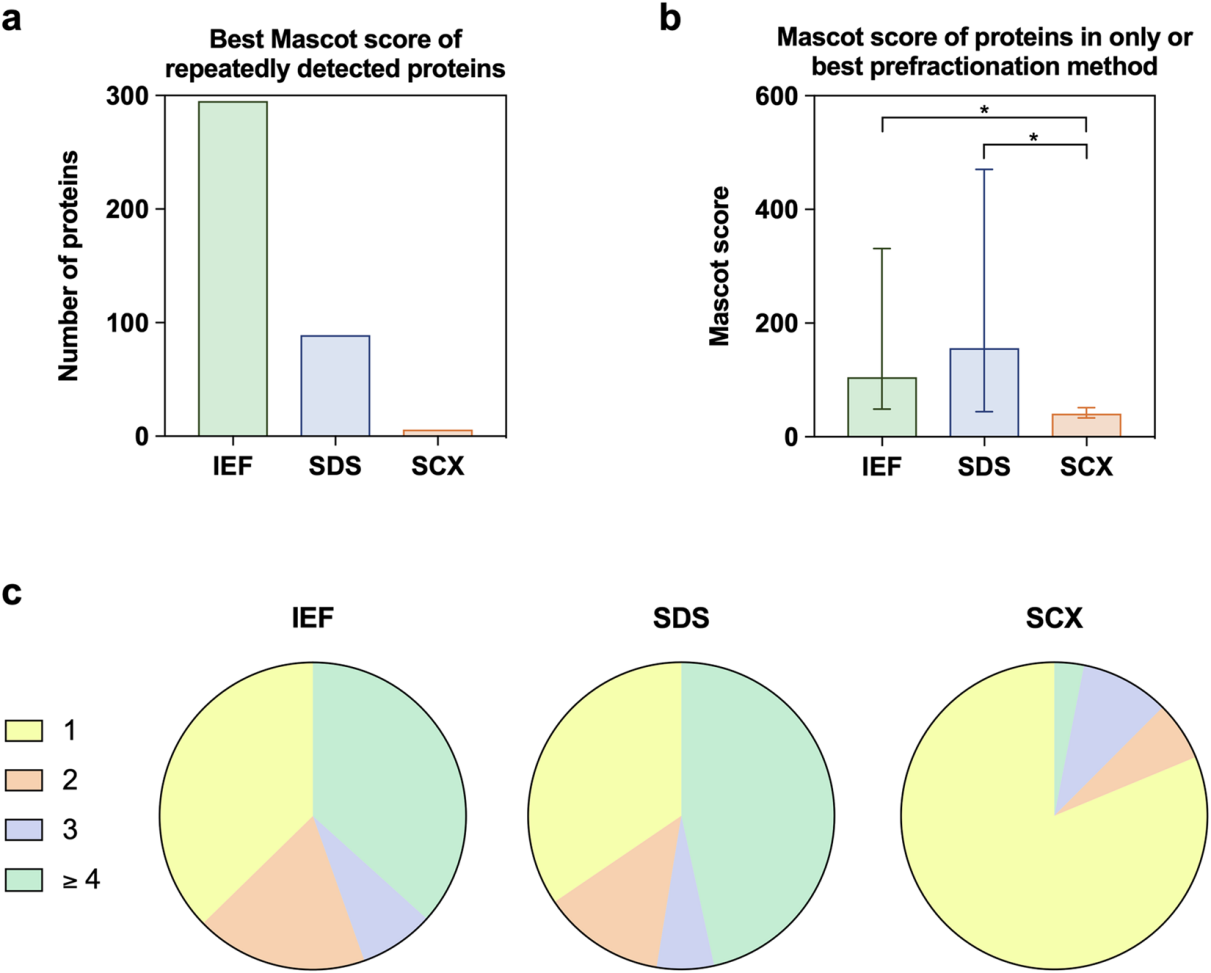
Analysis of human dentin proteome by mass spectrometry and combination of three different prefractionation methods. (**a**) Number of repeatedly detected proteins (390) with the best Mascot score in each prefractionation group. (**b**) Medians and 25–75% percentiles of Mascot scores achieved after IEF, SDS-PAGE and SCX. Each group contains proteins that were uniquely detected or provided the best Mascot score at repeated detection. Asterisks indicate significant differences between groups (*P* ≤ 0.05). (**c**) Pie charts show the number of single peptides detected in mass spectrometry by different prefractionation methods.

A comparison of the Mascot scores of all repeatedly detected proteins revealed that prefractionation by IEF led to the best score in 295 cases followed by SDS-PAGE (89) and SCX (9) (Fig. 2a). Likewise, the Mascot scores of proteins that were detected only or best by SCX were significantly lower compared to SDS-PAGE and IEF (Fig. 2b). IEF and SDS-PAGE enabled the detection of two or more peptides of each protein in over 60%, whereas only one peptide was found in 81% after SCX (Fig. 2c).

**Figure 3:**
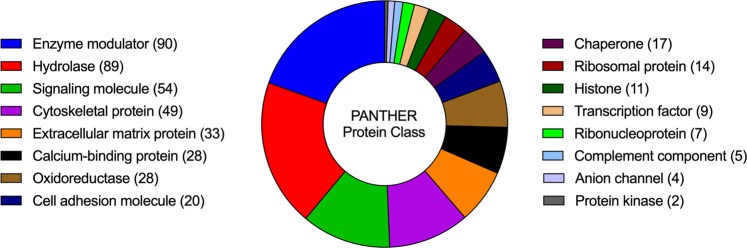
Protein classes detected in human dentin at the most general level and, in parentheses, the number of proteins in each family.

Protein classes at the most general level are illustrated in Fig. 3. The statistical overrepresentation test revealed 31 classes and subclasses to be enriched in comparison to the reference proteome. Table 1 shows all protein categories in hierarchical order from the highest to the lowest representation as well as respective *P*-values, which were significant in all cases (*P* ≤ 0.05). The enrichment fold changes reached up to 10.24 headed by the classes of complement components and chaperonins (Table 2). Heat shock protein 90 family chaperones followed with an overrepresentation of 8.88. However, metalloproteases and growth factors, which are typical components of dentin matrix, were located in the midfield with enrichments of 3.00- and 2.64-fold respectively. Underrepresented proteins were protein kinases (0.21) as well as transcription factors (0.13).

**Table 1:** Enrichment analysis by PANTHER with all detected classes or subclasses ordered by fold enrichment (number of contained proteins in parentheses) with their respective *P*-value and false discovery rate (FDR).

PANTHER protein class or subclass	Fold enrichment	*P*-value	FDR
Complement component (5)	10.24	3.54 × 10^−4^	3.17 × 10^−3^
Chaperonin (5)	10.24	3.54 × 10^−4^	3.05 × 10^−3^
Hsp90 family chaperone (3)	8.88	8.13 × 10^−3^	4.60 × 10^−2^
Extracellular matrix structural protein (10)	8.59	1.47 × 10^−6^	2.26 × 10^−5^
Serine protease inhibitor (30)	8.41	8.78 × 10^−17^	6.29 × 10^−15^
Tubulin (7)	8.11	7.77 × 10^−5^	7.96 × 10^−4^
Peroxidase (6)	6.66	6.21 × 10^−4^	4.95 × 10^−3^
Actin binding motor protein (6)	5.92	1.06 × 10^−3^	8.12 × 10^−3^
Anion channel (4)	5.61	8.70 × 10^−3^	4.80 × 10^−2^
Protease inhibitor (62)	5.23	9.68 × 10^−24^	2.08 × 10^−21^
Histone (11)	4.65	6.79 × 10^−5^	7.30 × 10^−4^
Extracellular matrix glycoprotein (17)	4.57	9.42 × 10^−7^	1.69 × 10^−5^
Actin and actin related protein (5)	4.30	9.11 × 10^−3^	4.90 × 10^−2^
Chaperone (17)	4.27	2.17 × 10^−6^	2.91 × 10^−5^
Extracellular matrix protein (33)	4.19	6.54 × 10^−11^	2.81 × 10^−9^
Ribonucleoprotein (7)	3.97	3.19 × 10^−3^	2.14 × 10^−2^
Intracellular calcium-sensing protein (20)	3.31	9.59 × 10^−6^	1.15 × 10^−4^
Calcium-binding protein (28)	3.29	1.93 × 10^−7^	4.61 × 10^−6^
Actin family cytoskeletal protein (28)	3.24	2.46 × 10^−7^	5.29 × 10^−6^
Annexin (11)	3.22	1.16 × 10^−3^	8.60 × 10^−3^
Metalloprotease (23)	3.00	9.08 × 10^−6^	1.15 × 10^−4^
Cell adhesion molecule (20)	2.75	1.07 × 10^−4^	1.04 × 10^−3^
Growth factor (12)	2.64	3.27 × 10^−3^	2.13 × 10^−2^
Cytoskeletal protein (49)	2.63	6.86 × 10^−9^	1.84 × 10^−7^
Serine protease (24)	2.48	1.29 × 10^−4^	1.21 × 10^−3^
Ribosomal protein (14)	2.39	5.46 × 10^−3^	3.46 × 10^−2^
Enzyme modulator (90)	2.26	5.81 × 10^−12^	3.12 × 10^−10^
Protease (66)	2.26	4.45 × 10^−9^	1.37 × 10^−7^
Signaling molecule (54)	2.12	8.89 × 10^−7^	1.74 × 10^−5^
Oxidoreductase (28)	1.72	7.61 × 10^−3^	4.55 × 10^−2^
Hydrolase (89)	1.72	1.45 × 10^−6^	2.41 × 10^−5^
Protein kinase (2)	0.21	6.93 × 10^−3^	4.26 × 10^−2^
Transcription factor (9)	0.21	1.22 × 10^−9^	4.39 × 10^−8^
Non-receptor serine/threonine protein kinase (1)	0.13	7.74 × 10^−3^	4.50 × 10^−2^

**Table 2:** Detailed list of the three most overrepresented protein classes or subclasses in human dentin with the detected proteins.

PANTHER protein class or subclass	Protein	UniProtKB entry	Mascot score
Complement component (10.24 fold)	Complement C3	CO3_HUMAN	2880.5
	Alpha-2-macroglobulin	A2MG_HUMAN	2761.1
	Complement C4-A	CO4A_HUMAN	707.8
	Complement C4-B	CO4B_HUMAN	277.6
	Complement C5	CO5_HUMAN	46.9
Chaperonin (10.24 fold)	10 kDa heat shock protein, mitochondrial	CH10_HUMAN	187.0
	T-complex protein 1 subunit theta	TCPQ_HUMAN	161.6
	T-complex protein 1 subunit beta	TCPB_HUMAN	67.5
	T-complex protein 1 subunit zeta	TCPZ_HUMAN	66.5
	T-complex protein 1 subunit alpha	TCPA_HUMAN	33.2
Hsp90 family chaperone (8.88 fold)	Endoplasmin	ENPL_HUMAN	2562.4
	Heat shock protein HSP 90-beta	HS90B_HUMAN	863.4
	Heat shock protein HSP 90-alpha	HS90A_HUMAN	743.5

## Discussion

In this study we analyzed the human dentin proteome by shotgun proteomics with three prefractionation methods: SDS-PAGE, SCX and IEF. 813 proteins were detected by combination of different separation strategies with high confidence, which exceeded previous studies^10–12^. The first study that analyzed the human dentin proteome was conducted by Park *et al*. in 2009 using SDS-PAGE^11^. Whereas other groups worked e.g. with demineralized dentin, pulverized tooth roots or extensive extraction times^10–12^, we chose a protocol which allows for quick processing and a high final protein concentration^6^, both of which is important in context of protein stability and detectability by LC-MS/MS.

So far, SDS-PAGE has been the primary fractionation technique in dentin proteomics^10–12^. A comparison of the obtained results from SDS-PAGE with previous studies revealed coincided as well as newly found proteins. However, it has to be considered that former proteomic approaches did not only use varying protein isolation techniques, but also searched other databases and applied different criteria for confidentiality such as >95% probability according to Peptide Prophet algorithm or the two-peptide rule^10–12,15^. Taking this into account, quantity of proteins detected after SDS-PAGE was comparable to previous studies^11^. In addition to this prefractionation method, we utilized SCX and IEF to simplify the complex protein mixture and successfully maximized the protein coverage.

From 813 proteins discovered by LC-MS/MS almost half (390) were detected by all three prefractionations with high confidence. Nevertheless, IEF enabled the detection of 327 unique proteins, SDS-PAGE and SCX contributed with 68 and 28 proteins respectively. Considering repeatedly detected proteins, IEF was once more the superior prefractionation method and led to the highest Mascot scores in over 75%. SCX, instead, performed poorly regarding the number of detected peptides as well as the Mascot scores in comparison to IEF and SDS-PAGE. Several studies compared these and other fractionation methods before and found IEF to be advantageous^14,16–18^, however, the choice of the best method seemed to be highly sample-dependent. In summary, the protein coverage by combination of IEF, SDS-PAGE and SCX appeared comprehensive and beneficial for human dentin. Protein expression within dentin matrix needs to be further validated by classical molecular biological methods in future projects.

Resulting proteins were classified by the PANTHER system^19^, an organized database located in the Gene Ontology Consortium. Protein classes and subclasses are established on basis of their phylogeny and function by computerized algorithms. Assignment of all proteins to PANTHER protein classes enabled structured data organization as well as effective navigation through the data set. Furthermore, we compared our protein list to a reference list to determine over- or underrepresentation of classes. Here, the reference proteome functions as a landmark, it has been defined by UniProt based on various criteria to represent a cross section of the taxonomic diversity within UniProtKB (23,381 proteins). Of all 33 reported protein classes and subclasses, 30 were significantly overrepresented (10.24-fold to 1.72-fold) and only 3 underrepresented (0.21-fold to 0.13-fold). However, the top 3 of overrepresented protein classes were complement components, chaperonins and Hsp90 family chaperones, all of which are involved in pulp inflammation and cellular stress response.

As part of the innate and adaptive immunity, complement components have various functions throughout the organism such as clearance of microbes, promotion of inflammation, recruitment of immune cells and lyse of pathogens^20^. In coordination with other immunological systems, the cascade is responsible for the resolution of inflammation and thus facilitates repair of diseased tissue. The anaphylatoxin C3a, which is a cleavage product of C3 and was detected in dentin, is known to induce migration of immune but also non-immune cells. C3a has been described to mobilize dental pulp stem cells and guide them to the affected tissue region^20,21^. Likewise, C5a recruits leukocytes and pulp progenitor cells and acts vasodilatative during inflammatory processes^22^. Furthermore, complement components of the extracellular dentin matrix or their respective derivates (C3a, C4a, and C5a) can form a cytolytic complex (MAC), attacking and lysing susceptible pathogen membranes^20^.

Beyond that, dentin matrix comprised several T-complex protein 1 (TCP1) subunits, which are members of the chaperonin containing TCP1 complex (CCT). This macromolecular chaperonin complex prevents misfolding or unfolds misfolded conformations of 10% of the whole cell proteome^23^, however, CCT has recently been described to regulate NF-κB transcription and to play a role in resolution of inflammation^24^.

Similar to chaperonins, the molecular chaperones of the heat shock protein 90 (Hsp90) family were overrepresented in human dentin and regulate proteostasis under stress conditions such as sudden temperature increases or inflammation. Not only do they conserve proteins in general, but they selectively maintain the structure and function of the cytosolic 26S proteasomes^25^. Whether in cytosol or circulating in extracellular space, proteasomes are functionally involved in inflammatory reactions by elimination of unfolded proteins or protein debris^26^. Furthermore, the detected endoplasmin (a.k.a. gp96) has been shown to activate the TLR2/4 pathway on regulatory T-cells via a C-terminal loop structure^27,28^ and might function as ligand for toll-like receptors in dental pulp^29^.

In conclusion, our study comprehensively covered the proteome of human dentin matrix by combination of different prefractionations prior LC-MS/MS. Isoelectric focusing turned out to be the most beneficial fractionation method and was superior to SDS-PAGE or SCX. Overall, immune-relevant proteins were found highly represented in dentin, which opens up many questions about the distinct role of dentin matrix proteins in pulp pathology as well as their potential to modulate inflammation.

## Methods

### Protein Isolation

Freshly extracted and caries-free human third molars (50) from donors aged 15 to 25 were collected with informed consent of the patients or a parent/legal guardian if they were under the age of 18. Sample collection was performed in accordance with the relevant guidelines and regulations, and approved by the Ethics Committee (Faculty of Medicine, University of Regensburg, Regensburg, Germany). Proteins were isolated from powdered dentin by 10% EDTA (269 mmol/l; EDTA Disodium Salt Dihydrate, AppliChem, Darmstadt, Germany) according to a previously described protocol^6^, concentrated by use of centrifugal filters with a molecular weight cutoff of 3,000 Da (Amicon^®^ Ultra-15 3K, Merck Millipore, Billerica, MA, USA) and transferred into phosphate buffered saline.

### SDS-PAGE and In-gel Digestion

50 µg dentin protein was separated on a 10% Bis-Tris protein gel (1.0 mm, 10-well NuPAGE™, Thermo Fisher Scientific, Waltham, MA, USA) using a MOPS buffer system and stained with SimplyBlue™ SafeStain (Thermo Fisher Scientific). The gel lane was cut into 20 consecutive slices, which were transferred into 2 ml microtubes and washed with 50 mM NH_4_HCO_3_, 50 mM NH_4_HCO_3_/acetonitrile (3:1) and 50 mM NH_4_HCO_3_/acetonitrile (1:1). Gel pieces were lyophilized after shrinking by 100% acetonitrile, reduced with DTT at 57 °C for 30 min to block cysteines, and alkylated with iodoacetamide at room temperature in the dark for 30 min. Gel slices were washed and lyophilized again, and subjected to in-gel tryptic digest at 37 °C with 2 µg trypsin (Promega™ Trypsin Gold Mass Spectrometry Grade, Thermo Fisher Scientific) per 100 µl gel overnight. Peptides were eluted twice with 100 mM NH_4_HCO_3_ followed by an extraction with 50 mM NH_4_HCO_3_ in 50% acetonitrile, and finally lyophilized.

### Filter-aided Sample Preparation (FASP)

Samples underwent FASP before IEF and SCX. Therefore, 150 µg dentin protein (25 µl) was added to 250 µl denaturation buffer (8 M urea/100 mM NH_4_HCO_3_/0.3% SDS) and incubated at 37 °C for 15 min. After incubation with 5 µg DTT at 37 °C for 30 min to reduce disulfide bridges, 25 µg iodoacetamide was added for cysteine alkylation and incubated at room temperature in the dark for 30 min. Subsequent to quenching with 25 µg DTT for 15 min, the solution was transferred to a Nanosep^®^ −30K-device equilibrated with 8 M Urea/100 mM NH_4_HCO_3_ (Nanosep^®^ 30K Omega™, Pall, Port Washington, NY, USA) and centrifuged at 10,000 rpm for 8 min. After three washing steps with 8 M urea/100 mM NH_4_HCO_3_ and one with 2 M urea/100 mM NH_4_HCO_3_, proteins were digested on the membrane with 100 µl of a 50 mM NH_4_HCO_3_ solution containing 2 µg trypsin in a wet chamber at 37 °C overnight. Resulting peptides were collected by centrifugation with 100 µl of 50 mM NH_4_HCO_3_ at 10,000 rpm for 5 min followed by 100 µl deionized water. Combined eluates were lyophilized.

### OFFGEL Isoelectric Focusing

The peptide mixture resulting from FASP was separated into 24 fractions on the Agilent 3100 OFFGEL fractionator (Agilent Technologies, Santa Clara, CA, USA). Dried peptides were resuspended in 3.6 ml isoelectric focusing buffer containing 5% glycerol and 1% ampholytes (GE Healthcare, Chicago, IL, USA). Immobilized pH gradient strips (Immobiline^®^ Drystrip pH 3–10 NL, 24 cm, GE Healthcare) were rehydrated with 40 µl buffer per well for 20 min and peptide solution (150 µl/well) was added. Soaked filter paper wicks were placed at the ends of the gel strips and overlaid with mineral oil to prevent drying. IEF was conducted at 4,500 V with a limiting current of 50 µA and a focusing target of 50 kVh at 20 °C overnight. Peptide fractions were transferred to low binding tubes (MAXYMum Recovery^®^, VWR, Radnor, PA, USA) and cleaned up by C18 stage tips (Thermo Fisher Scientific).

### Strong Cation Exchange Chromatography

Peptides from FASP were reconstituted in 30 µl of a 20% acetonitrile/0.4% formic acid solution and fractionated using stage tips SCX (Thermo Fisher Scientific). Initially, the SCX material was rinsed with 30 µl of 1 M NaCl/20% acetonitrile/0.4% formic acid, 30 µl of 20% acetonitrile/0.4% formic acid, four times 30 µl of acetonitrile, and 30 µl of 1 M NaCl/20% acetonitrile/0.4% formic acid. Before application of the sample, the SCX material was re-equilibrated two times in 30 µl of 20% acetonitrile/0.4% formic acid. The SCX stage tip was then washed twice with 20 µl sample buffer, and elution was facilitated by rinsing with 20 µl of each elution buffer twice. 20 peptide fractions were generated with an elution buffer series from 50 to 500 mM CH_3_CO_2_NH_4_ in 20% acetonitrile. C18 stage tip purification was performed to desalt the fractions before LC-MS.

### LC-MS/MS Analysis

Lyophilized peptides were reconstituted in 20 µl of 1% formic acid and processed by reversed-phase chromatography on an UltiMate™ 3000 RSLCnano System (Thermo Fisher Scientific) equipped with an Acclaim™ Pepmap™ 100 C18 preconcentration column (100 µm × 20 mm; Thermo Fisher Scientific) in front of an Acclaim™ Pepmap™ 100 C18 nano column (75 µm × 150 mm; Thermo Fisher Scientific). Peptides were separated by a linear gradient of 4 to 40% acetonitrile in 0.1% formic acid at a flow rate of 300 nl/min over 90 min and subjected to an on-line-coupled maXis™ plus UHR-QTOF system (Bruker, Billerica, MA, USA) via a CaptiveSpray nanoflow electrospray source (Bruker).

Data-dependent acquisition of MS/MS spectra by CID fragmentation was performed at a resolution of minimum 60,000 for MS and MS/MS scans. The MS spectra rate of the precursor scan was 2 Hz processing a mass range (m/z) between 175 and 2000. A dynamic method with a fixed cycle time (3 s) and an m/z-dependent collision energy adjustment (34 to 55 eV) was applied by the Compass 1.7 software (Bruker). Raw data was processed in Data Analysis 4.2, and Protein Scape 3.1.3 (Bruker) in connection with Mascot 2.5.1 (Matrix Science, Boston, MA, USA) searching of the Swiss-Prot database (*homo sapiens*, release-2016_7, 20198). The Swiss-Prot database contains over 550,000 human protein sequences that are permanently reviewed by an expert biocuration team to avoid redundant or identical detections^30^. Search parameters were as follows: enzyme specificity trypsin with 1 missed cleavage allowed, precursor tolerance 5 ppm, MS/MS tolerance 0.04 Da, carbamidomethylation or propionamide modification of cysteine, oxidation of methionine, deamidation of asparagine and glutamine were set as variable modifications. The Mascot score, which is a probability-based scoring that allows judgement about the validity of a protein match and statistical significance^31^, was cut off at 30 and search conditions were adjusted to provide a false discovery rate (FDR) of less than 1% to facilitate a confident protein identification. If necessary, fragment spectra were inspected manually.

### Data processing and statistical analysis

Results from three fractionation methods were merged to a table containing all proteins in alphabetical order (Supplementary Table S1). Besides the full protein name, the list contained the respective UniProtKB entry, the molecular weight and the isoelectric point. For repeatedly detected proteins, all fractionation methods that allowed for the detection of a specific protein (‘successful prefractionation methods’) as well as the ‘best prefractionation method’ based on its Mascot score were acquired. Furthermore, the Mascot score, the number of detected single peptides and the sequence coverage were enlisted for the best fractionation method.

Prefractionation methods were compared with one another based on the parameters named above. Data were treated nonparametrically and analyzed using the Mann–Whitney *U*-test on an α = 0.05 level of significance (GraphPad Prism 7; GraphPad Software, La Jolla, CA).

### Protein classification and overrepresentation analysis

UniProtKB entries were used as primary protein identifiers and submitted to the PANTHER (protein annotation through evolutionary relationship) protein classification system (version 13.1 released 2018/02/03). Since a protein can be classified according to more than one independent ontology, the number of hits in each class over the total number of class hits was determined^19^. Further details of all parent terms and subclasses are provided in Supplementary Table S2.

All proteins were compared to a reference proteome data set (*homo sapiens*) provided by PANTHER to determine whether a family or subfamily was under- or overrepresented^19^. Representation analysis was conducted online (http://www.pantherdb.org) by the Fisher’s Exact test with an FDR multiple test correction by the Benjamini-Hochberg procedure.

## Supplementary information

Supplementary Tables

## Data Availability

All underlying data is provided as Supplementary Information.
