# Supplementary material for: Shotgun Proteomics of Human Dentin with Different Prefractionation Methods
Source: Sci Rep. 2019 Mar 14;9:4457. doi: 10.1038/s41598-019-41144-x (PMC6418255; doi:10.1038/s41598-019-41144-x)
Supplement: Supplementary file 1 — Supplementary Tables [file 41598_2019_41144_MOESM1_ESM.pdf]

Supplementary Information

Shotgun Proteomics of Human Dentin with Different Prefractionation Methods

M. Widbiller[1], H. Schweikl[1], A. Bruckmann[2], A. Rosendahl[1], E. Hochmuth[2], S. R. Lindner[3], W. Buchalla[1], K. M. Galler[1]

[1]*Department of Conservative Dentistry and Periodontology, University Hospital Regensburg, Regensburg, Germany*
[2]*Biochemistry Center Regensburg, Laboratory for RNA Biology, University of Regensburg, Regensburg, Germany*
[3]*Department of Oral- and Maxillofacial Surgery, University Hospital Regensburg, Regensburg, Germany*

Supplementary Table S1 List of all proteins detected with confidence in human dentin by three prefractionation methods: isoelectric focusing (IEF), sodium dodecyl sulfate polyacrylamide gel electrophoresis (SDS) and strong cation exchange chromatography (SCX). For repeatedly detected proteins, all fractionation methods that allowed for detection (‘successful prefractionation methods’) as well as the ‘best prefractionation method’ based on its Mascot score were mentioned.

Protein	UniProtKB entry	Molecular weight [kDa]	Isoelectric point	Successful prefrationation methods	Best prefractionation method	Mascot score in best method	Number of peptides	Sequence coverage [%]
1-phosphatidylinositol 4,5-bisphosphate phosphodiesterase delta-1	PLCD1_HUMAN	85.6	6.2	SCX		34.4	1	1.3
10 kDa heat shock protein, mitochondrial	CH10_HUMAN	10.9	8.9	SCX, IEF	IEF	187.0	3	32.4
14-3-3 protein beta/alpha	1433B_HUMAN	28.1	4.8	SDS, IEF	IEF	208.2	5	19.9
14-3-3 protein epsilon	1433E_HUMAN	29.2	4.6	SDS, IEF	SDS	442.5	8	29.0
14-3-3 protein gamma	1433G_HUMAN	28.3	4.8	SDS		174.2	5	20.6
14-3-3 protein sigma	1433S_HUMAN	27.8	4.7	SDS		152.0	4	12.1
14-3-3 protein theta	1433T_HUMAN	27.7	4.7	SDS, IEF	SDS	291.8	6	27.3
14-3-3 protein zeta/delta	1433Z_HUMAN	27.7	4.7	SCX, SDS, IEF	IEF	425.6	6	27.3
15 kDa selenoprotein	SEP15_HUMAN	17.7	5.0	SCX, SDS, IEF	SDS	52.6	1	9.9
2'-deoxynucleoside 5'-phosphate N-hydrolase 1	DNPH1_HUMAN	19.1	5.0	IEF		65.3	1	9.8
26S proteasome non-ATPase regulatory subunit 12	PSD12_HUMAN	52.9	7.5	IEF		35.5	1	4.6
26S proteasome non-ATPase regulatory subunit 3	PSMD3_HUMAN	60.9	8.5	IEF		54.2	1	2.8
26S proteasome non-ATPase regulatory subunit 6	PSMD6_HUMAN	45.5	5.5	IEF		69.9	1	3.9
40S ribosomal protein S20	RS20_HUMAN	13.4	9.9	IEF		50.5	1	9.2
40S ribosomal protein S24	RS24_HUMAN	15.4	10.8	IEF		56.4	1	9.0
40S ribosomal protein S3	RS3_HUMAN	26.7	9.7	IEF		46.5	1	3.7
40S ribosomal protein S3a	RS3A_HUMAN	29.9	9.8	IEF		68.4	1	6.4
40S ribosomal protein S7	RS7_HUMAN	22.1	10.1	IEF		46.9	1	6.2
5'-nucleotidase	5NTD_HUMAN	63.3	6.6	IEF		81.3	2	5.1
6-phosphogluconolactonase	6PGL_HUMAN	27.5	5.7	SDS, IEF	SDS	130.7	3	16.3
60 kDa heat shock protein, mitochondrial	CH60_HUMAN	61.0	5.7	IEF		69.3	1	3.1
60 kDa SS-A/Ro ribonucleoprotein	RO60_HUMAN	60.6	8.3	IEF		46.5	1	2.0
60S acidic ribosomal protein P0	RLA0_HUMAN	34.3	5.7	IEF		82.4	2	6.9
60S acidic ribosomal protein P1	RLA1_HUMAN	11.5	4.2	IEF		38.9	1	14.0
60S acidic ribosomal protein P2	RLA2_HUMAN	11.7	4.4	IEF		136.5	2	27.0
60S ribosomal protein L12	RL12_HUMAN	17.8	9.5	IEF		39.5	1	5.5
60S ribosomal protein L14	RL14_HUMAN	23.4	10.9	IEF		43.8	1	5.6
60S ribosomal protein L15	RL15_HUMAN	24.1	11.6	IEF		47.9	1	5.9
60S ribosomal protein L27	RL27_HUMAN	15.8	10.6	IEF		48.4	1	5.9
60S ribosomal protein L5	RL5_HUMAN	34.3	9.7	IEF		34.3	1	6.1
60S ribosomal protein L6	RL6_HUMAN	32.7	10.6	IEF		169.2	2	11.1
72 kDa type IV collagenase	MMP2_HUMAN	73.8	5.3	SCX, SDS, IEF	IEF	552.2	10	15.6
78 kDa glucose-regulated protein	GRP78_HUMAN	72.3	5.1	SCX, SDS, IEF	IEF	2327.6	35	35.3
A disintegrin and metalloproteinase with thrombospondin motifs 8	ATS8_HUMAN	96.4	5.8	IEF		99.6	2	4.7
Acetyl-CoA acetyltransferase, cytosolic	THIC_HUMAN	41.3	6.5	IEF		43.6	1	2.8
Acetyl-CoA acetyltransferase, mitochondrial	THIL_HUMAN	45.2	9.0	IEF		32.9	1	4.0
Acid ceramidase	ASAH1_HUMAN	44.6	7.5	IEF		119.3	2	8.9
Acid sphingomyelinase-like phosphodiesterase 3b	ASM3B_HUMAN	50.8	5.4	IEF		54.9	1	2.6
Acidic leucine-rich nuclear phosphoprotein 32 family member A	AN32A_HUMAN	28.6	4.0	SDS, IEF	SDS	161.3	3	16.1
Acidic leucine-rich nuclear phosphoprotein 32 family member B	AN32B_HUMAN	28.8	3.9	IEF		59.7	2	10.8
Actin-related protein 2	ARP2_HUMAN	44.7	6.3	IEF		123.0	3	10.9
Actin-related protein 2/3 complex subunit 2	ARPC2_HUMAN	34.3	6.8	IEF		48.6	1	3.7
Actin-related protein 2/3 complex subunit 3	ARPC3_HUMAN	20.5	8.8	SCX, IEF	IEF	50.3	1	6.2
Actin-related protein 2/3 complex subunit 4	ARPC4_HUMAN	19.7	8.5	SDS, IEF	IEF	110.0	3	16.1
Actin-related protein 2/3 complex subunit 5	ARPC5_HUMAN	16.3	5.5	IEF		32.6	1	7.9
Actin-related protein 2/3 complex subunit 5-like protein	ARP5L_HUMAN	16.9	6.2	IEF		41.6	1	7.8
Actin-related protein 3C	ARP3C_HUMAN	23.7	5.4	SDS		38.3	1	5.2
Actin, alpha cardiac muscle 1	ACTC_HUMAN	42.0	5.2	IEF		1203.9	15	36.6
Actin, cytoplasmic 1	ACTB_HUMAN	41.7	5.3	SCX, SDS, IEF	IEF	1952.1	26	66.1
Actin, gamma-enteric smooth muscle	ACTH_HUMAN	41.8	5.3	SDS		332.3	6	14.4
Activated RNA polymerase II transcriptional coactivator p15	TCP4_HUMAN	14.4	9.6	IEF		61.1	1	8.7
Acyl-CoA-binding protein	ACBP_HUMAN	10.0	6.1	IEF		135.9	3	52.9
ADAMTS-like protein 2	ATL2_HUMAN	104.6	6.0	IEF		44.2	1	2.2
Adapter molecule crk	CRK_HUMAN	33.8	5.4	IEF		37.7	1	3.6
Adenosylhomocysteinase	SAHH2_HUMAN	47.7	5.9	IEF		35.4	1	1.9

Adenosylhomocysteinase 2	SAHH_HUMAN	58.9	6.5	IEF		52.0	1	2.3
Adenylyl cyclase-associated protein 1	CAP1_HUMAN	51.9	8.2	SDS, IEF	IEF	201.2	3	11.4
Adipocyte enhancer-binding protein 1	AEBP1_HUMAN	130.8	5.0	SDS, IEF	IEF	408.7	9	7.9
ADP-ribosylation factor 5	ARF5_HUMAN	20.5	6.3	IEF		61.8	2	11.7
Adseverin	ADSV_HUMAN	80.4	5.5	SDS, IEF	IEF	211.4	5	11.3
Afamin	AFAM_HUMAN	69.0	5.6	SCX, SDS, IEF	SDS	177.5	4	8.8
Agrin	AGRIN_HUMAN	217.1	6.0	IEF		42.9	1	0.5
Alcohol dehydrogenase [NADP(+)]	AK1A1_HUMAN	36.5	6.3	SCX		87.1	2	6.8
Alcohol dehydrogenase class-3	ADHX_HUMAN	39.7	7.5	IEF		46.7	1	2.4
Aldehyde dehydrogenase family 1 member A3	AL1A3_HUMAN	56.1	7.0	IEF		37.4	1	2.1
Aldose reductase	ALDR_HUMAN	35.8	6.5	IEF		37.3	1	2.5
Alkaline phosphatase, tissue-nonspecific isozyme	PPBT_HUMAN	57.3	6.2	SCX, SDS, IEF	IEF	176.3	3	12.2
Alpha-1-acid glycoprotein 1	A1AG1_HUMAN	23.5	4.9	SCX, SDS, IEF	IEF	492.8	8	31.8
Alpha-1-acid glycoprotein 2	A1AG2_HUMAN	23.6	5.0	SCX, SDS, IEF	IEF	261.0	4	30.8
Alpha-1-antichymotrypsin	AACT_HUMAN	47.6	5.3	SCX, SDS, IEF	IEF	654.3	10	30.3
Alpha-1-antitrypsin	A1AT_HUMAN	46.7	5.4	SCX, SDS, IEF	IEF	1758.0	21	47.1
Alpha-1B-glycoprotein	A1BG_HUMAN	54.2	5.6	SCX, SDS, IEF	IEF	365.4	8	19.8
Alpha-2-antiplasmin	A2AP_HUMAN	54.5	5.9	IEF		188.8	4	13.6
Alpha-2-HS-glycoprotein	FETUA_HUMAN	39.3	5.4	SCX, SDS, IEF	IEF	7006.6	58	68.1
Alpha-2-macroglobulin	A2MG_HUMAN	163.2	6.0	SCX, SDS, IEF	IEF	2761.1	45	35.1
Alpha-actinin-1	ACTN1_HUMAN	103.0	5.2	IEF		464.8	9	11.9
Alpha-actinin-4	ACTN4_HUMAN	104.8	5.3	SCX, SDS, IEF	IEF	532.0	11	15.6
Alpha-enolase	ENOA_HUMAN	47.1	7.0	SCX, SDS, IEF	IEF	647.3	10	33.9
Alpha-L-iduronidase	IDUA_HUMAN	72.6	9.2	IEF		51.0	1	2.0
Alpha-mannosidase 2	MA2A1_HUMAN	131.1	7.2	IEF		58.6	1	1.0
Ameloblastin	AMBN_HUMAN	48.3	4.8	SDS, IEF	SDS	41.0	1	2.0
Aminopeptidase B	AMPB_HUMAN	72.5	5.5	SCX		34.8	1	2.8
Aminopeptidase N	AMPN_HUMAN	109.5	5.3	IEF		31.0	1	1.6
Ammonium transporter Rh type B	RHBG_HUMAN	47.2	8.7	SDS		37.4	1	2.3
Amyloid beta A4 protein	A4_HUMAN	86.9	4.7	SCX, SDS, IEF	IEF	1378.1	22	21.2
Amyloid-like protein 2	APLP2_HUMAN	86.9	4.7	SCX, SDS, IEF	IEF	342.8	6	10.9
Angiogenin	ANGI_HUMAN	16.5	9.7	SCX, SDS, IEF	SDS	701.0	9	47.6
Angiotensinogen	ANGT_HUMAN	53.1	5.9	SCX, SDS, IEF	SDS	367.8	5	17.1
Annexin A1	ANXA1_HUMAN	38.7	6.6	SCX, SDS, IEF	IEF	1175.5	15	46.0
Annexin A2	ANXA2_HUMAN	38.6	7.6	SCX, SDS, IEF	SDS	1208.6	17	41.9
Annexin A4	ANXA4_HUMAN	35.9	5.8	SCX, SDS, IEF	SDS	516.9	9	28.2
Annexin A5	ANXA5_HUMAN	35.9	4.9	SCX, SDS, IEF	SDS	1264.1	20	50.3
Annexin A6	ANXA6_HUMAN	75.8	5.4	SCX, SDS, IEF	IEF	589.1	12	25.3
Antileukoproteinase	SLPI_HUMAN	14.3	9.1	SDS, IEF	IEF	60.5	1	9.1
Antithrombin-III	ANT3_HUMAN	52.6	6.3	SCX, SDS, IEF	IEF	4113.0	51	64.2
AP-2 complex subunit beta	AP2B1_HUMAN	104.5	5.2	IEF		53.9	1	1.6
Apolipoprotein A-I	APOA1_HUMAN	30.8	5.6	SCX, SDS, IEF	IEF	1317.7	22	54.3
Apolipoprotein A-II	APOA2_HUMAN	11.2	6.3	IEF		50.9	1	20.0
Apolipoprotein A-IV	APOA4_HUMAN	45.4	5.3	SCX, SDS, IEF	IEF	2139.8	31	43.7
Apolipoprotein C-I	APOC1_HUMAN	9.3	8.0	SCX, IEF	IEF	55.2	2	26.5
Apolipoprotein C-III	APOC3_HUMAN	10.8	5.2	SDS, IEF	IEF	84.4	1	16.2
Apolipoprotein E	APOE_HUMAN	36.1	5.6	SDS, IEF	IEF	137.0	4	14.5
Arginase-1	ARGI1_HUMAN	34.7	6.7	SDS, IEF	IEF	92.2	2	9.9
Aspartate aminotransferase, cytoplasmic	AATC_HUMAN	46.2	6.5	SDS		31.8	1	3.4
Aspartate aminotransferase, mitochondrial	AATM_HUMAN	47.5	9.1	IEF		152.8	3	6.3
Aspartyl/asparaginyl beta-hydroxylase	ASPH_HUMAN	85.8	4.9	SCX, SDS, IEF	IEF	386.8	8	14.9
Asporin	ASPN_HUMAN	43.4	6.6	SCX, SDS, IEF	IEF	250.8	5	20.3
Astrocytic phosphoprotein PEA-15	PEA15_HUMAN	15.0	4.9	IEF		31.4	1	13.1
ATP synthase subunit alpha, mitochondrial	ATPA_HUMAN	59.7	9.2	SDS		38.6	1	1.8
ATP synthase subunit beta, mitochondrial	ATPB_HUMAN	56.5	5.3	IEF		145.7	3	7.9
ATP synthase subunit O, mitochondrial	ATPO_HUMAN	23.3	10.0	IEF		42.8	1	8.0
ATP-dependent 6-phosphofructokinase, liver type	PFKAL_HUMAN	85.0	7.3	IEF		56.6	1	1.7
ATP-dependent RNA helicase A	DHX9_HUMAN	140.9	6.4	IEF		110.8	3	3.4
ATP-dependent RNA helicase DDX3X	DDX3X_HUMAN	73.2	6.7	IEF		39.6	1	3.3
ATPase family AAA domain-containing protein 3C	ATD3C_HUMAN	46.4	9.4	IEF		36.3	1	3.4
Axin interactor, dorsalization-associated protein	AIDA_HUMAN	35.0	6.1	IEF		39.8	1	2.6
Basement membrane-specific heparan sulfate proteoglycan core protein	PGBM_HUMAN	468.5	6.1	SCX, SDS, IEF	IEF	1283.0	22	6.6
Beta-2-glycoprotein 1	APOH_HUMAN	38.3	8.3	SDS, IEF	IEF	444.8	6	26.1
Beta-2-microglobulin	B2MG_HUMAN	13.7	6.1	IEF		311.7	4	21.8

Biglycan	PGS1_HUMAN	41.6	7.2	SCX, SDS, IEF	IEF	7932.5	79	66.6
Bone morphogenetic protein 8B	BMP8B_HUMAN	44.7	8.8	SCX, IEF	IEF	77.8	2	6.0
Bone sialoprotein 2	SIAL_HUMAN	35.1	4.1	SCX, IEF	IEF	42.8	1	4.1
Breast cancer type 2 susceptibility protein	BRCA2_HUMAN	384.0	6.3	IEF		32.9	1	0.2
BTB/POZ domain-containing protein KCTD12	KCD12_HUMAN	35.7	5.5	SCX, SDS, IEF	SDS	176.1	5	18.8
C-C motif chemokine 14	CCL14_HUMAN	10.7	8.9	IEF		102.1	2	41.9
C-reactive protein	CRP_HUMAN	25.0	5.5	SCX, SDS, IEF	SDS	287.1	5	21.0
C-type lectin domain family 11 member A	CLC11_HUMAN	35.7	5.1	SCX, SDS, IEF	IEF	1010.2	17	35.3
C-type mannose receptor 2	MRC2_HUMAN	166.6	5.5	SCX, IEF	IEF	122.7	2	1.8
Cadherin-1	CADH1_HUMAN	97.4	4.6	IEF		57.1	1	1.0
Cadherin-2	CADH2_HUMAN	99.7	4.6	IEF		65.7	1	1.4
Calbindin	CALB1_HUMAN	30.0	4.7	SDS, IEF	IEF	102.5	2	9.2
Calcium-binding protein 39-like	CB39L_HUMAN	39.1	8.5	SDS, IEF	IEF	37.6	1	2.7
Calmodulin	CALM_HUMAN	16.8	4.1	SCX, SDS, IEF	IEF	252.1	3	30.9
Calmodulin-like protein 3	CALL3_HUMAN	16.9	4.3	SDS		53.9	1	11.4
Calmodulin-like protein 5	CALL5_HUMAN	15.9	4.3	SDS		31.0	1	15.8
Calnexin	CALX_HUMAN	67.5	4.5	IEF		213.0	4	10.6
Calpain small subunit 1	CPNS1_HUMAN	28.3	5.0	SDS		33.2	1	3.7
Calpain-2 catalytic subunit	CAN2_HUMAN	79.9	4.9	SCX, IEF	IEF	43.4	1	2.1
Calreticulin	CALR_HUMAN	48.1	4.3	SCX, SDS, IEF	IEF	1154.5	14	46.3
Calsequestrin-1	CASQ1_HUMAN	45.1	4.0	IEF		65.2	2	8.3
Calsequestrin-2	CASQ2_HUMAN	46.4	4.2	SDS, IEF	IEF	178.7	3	10.3
cAMP-dependent protein kinase type II-alpha regulatory subunit	KAP2_HUMAN	45.5	5.0	SCX		34.8	1	3.7
Carbonic anhydrase 1	CAH1_HUMAN	28.9	6.6	SCX, SDS, IEF	SDS	273.5	4	28.0
Carbonic anhydrase 2	CAH2_HUMAN	29.2	6.9	SDS, IEF	SDS	100.0	3	15.4
Carbonyl reductase [NADPH] 1	CBR1_HUMAN	30.4	8.6	IEF		113.9	3	13.4
Carboxypeptidase B2	CBPB2_HUMAN	48.4	7.6	SDS, IEF	IEF	78.9	1	2.8
Carboxypeptidase E	CBPE_HUMAN	53.1	5.0	IEF		65.1	1	4.6
Carboxypeptidase Q	CBPQ_HUMAN	51.9	5.8	IEF		38.5	1	2.3
Carboxypeptidase Z	CBPZ_HUMAN	73.6	8.2	IEF		35.6	1	1.5
Cartilage oligomeric matrix protein	COMP_HUMAN	82.8	4.4	IEF		31.3	1	1.7
Cartilage-associated protein	CRTAP_HUMAN	46.5	5.5	IEF		148.8	3	10.0
Casein kinase II subunit alpha	CSK21_HUMAN	45.1	7.3	IEF		31.2	1	1.8
Catenin beta-1	CTNB1_HUMAN	85.4	5.5	IEF		66.8	1	1.8
Cathepsin L1	CATL1_HUMAN	37.5	5.3	SDS, IEF	IEF	85.2	2	3.6
Cathepsin O	CATO_HUMAN	35.9	7.1	SCX, SDS, IEF	SDS	65.3	2	7.8
Caveolin-1	CAV1_HUMAN	20.5	5.6	SDS		39.7	1	11.8
CD276 antigen	CD276_HUMAN	57.2	4.8	IEF		31.4	1	3.4
CD44 antigen	CD44_HUMAN	81.5	5.1	SCX		40.7	1	1.6
CD5 antigen-like	CD5L_HUMAN	38.1	5.3	SDS, IEF	IEF	188.8	4	12.4
CD9 antigen	CD9_HUMAN	25.4	6.8	IEF		63.1	1	4.4
Cellular retinoic acid-binding protein 1	RABP1_HUMAN	15.6	5.3	SCX, SDS, IEF	IEF	244.2	4	37.2
Ceruloplasmin	CERU_HUMAN	122.1	5.4	SCX, SDS, IEF	IEF	588.0	11	16.1
Chitinase domain-containing protein 1	CHID1_HUMAN	44.9	8.7	SCX, SDS, IEF	IEF	700.0	13	38.9
Chloride intracellular channel protein 1	CLIC1_HUMAN	26.9	5.1	IEF		45.4	1	3.7
Chondroadherin	CHAD_HUMAN	40.5	9.5	SCX, SDS, IEF	IEF	1927.0	28	47.9
Chondroitin sulfate proteoglycan 4	CSPG4_HUMAN	250.4	5.3	IEF		89.7	2	1.0
Chromodomain-helicase-DNA-binding protein 8	CHD8_HUMAN	290.3	6.0	IEF		42.5	1	0.3
Chromogranin-A	CMGA_HUMAN	50.7	4.6	IEF		154.2	2	7.9
Clathrin heavy chain 1	CLH1_HUMAN	191.5	5.5	IEF		194.1	3	3.1
Clusterin	CLUS_HUMAN	52.5	5.9	SCX, SDS, IEF	IEF	1475.6	23	32.1
Coagulation factor IX	FA9_HUMAN	51.7	5.3	SCX, SDS, IEF	IEF	2407.8	34	46.2
Coagulation factor VII	FA7_HUMAN	51.6	6.9	SCX, SDS, IEF	IEF	1034.0	12	23.8
Coagulation factor X	FA10_HUMAN	54.7	5.7	SCX, SDS, IEF	IEF	1680.3	26	28.3
Coagulation factor XII	FA12_HUMAN	67.7	8.0	IEF		104.2	1	2.9
Coatomer subunit beta	COPB_HUMAN	107.1	5.7	IEF		73.2	2	2.2
Cofilin-1	COF1_HUMAN	18.5	8.2	SDS, IEF	IEF	209.8	5	48.8
Coiled-coil domain-containing protein 80	CCD80_HUMAN	108.1	9.7	SCX, SDS, IEF	IEF	220.5	5	6.0
Collagen alpha-1(I) chain	CO1A1_HUMAN	138.9	5.6	SCX, SDS, IEF	SDS	2707.8	44	20.5
Collagen alpha-1(II) chain	CO2A1_HUMAN	141.7	6.6	SDS, IEF	IEF	125.9	3	3.3
Collagen alpha-1(V) chain	CO5A1_HUMAN	183.4	4.9	SCX, SDS, IEF	IEF	842.6	11	8.5
Collagen alpha-1(VI) chain	CO6A1_HUMAN	108.5	5.3	SCX, IEF	IEF	244.9	6	7.9
Collagen alpha-1(XI) chain	COBA1_HUMAN	181.0	5.1	SCX, SDS, IEF	IEF	647.6	10	5.5
Collagen alpha-1(XII) chain	COCA1_HUMAN	332.9	5.4	SCX, SDS, IEF	IEF	1971.0	41	16.7

Collagen alpha-1(XV) chain	COFA1_HUMAN	141.6	4.9	SCX		46.6	1	0.9
Collagen alpha-1(XVI) chain	COGA1_HUMAN	157.7	8.1	IEF		38.6	1	1.2
Collagen alpha-1(XVIII) chain	COIA1_HUMAN	178.1	5.7	SCX, SDS, IEF	IEF	206.9	4	3.1
Collagen alpha-1(XXII) chain	COMA1_HUMAN	161.0	6.9	SDS, IEF	IEF	125.0	2	2.2
Collagen alpha-2(I) chain	CO1A2_HUMAN	129.2	9.1	SCX, SDS, IEF	IEF	3627.1	48	20.2
Collagen alpha-2(V) chain	CO5A2_HUMAN	144.8	6.1	SCX, SDS, IEF	IEF	462.9	7	6.7
Collagen alpha-2(VI) chain	CO6A2_HUMAN	108.5	5.9	IEF		103.0	3	3.4
Collagen alpha-2(XI) chain	COBA2_HUMAN	171.7	5.9	SCX, SDS, IEF	SDS	2467.1	36	11.8
Collagen alpha-3(V) chain	CO5A3_HUMAN	172.0	6.4	SCX		51.2	1	1.0
Collagen alpha-3(VI) chain	CO6A3_HUMAN	343.5	6.3	SCX, SDS, IEF	IEF	1354.8	28	12.2
Collagen triple helix repeat-containing protein 1	CTHR1_HUMAN	26.2	8.3	SCX, SDS, IEF	SDS	212.6	5	22.6
Collagenase 3	MMP13_HUMAN	53.8	5.3	IEF		30.2	1	3.2
Complement C1q subcomponent subunit A	C1QA_HUMAN	26.0	9.3	IEF		34.4	1	4.5
Complement C1q subcomponent subunit B	C1QB_HUMAN	26.7	8.8	SDS, IEF	SDS	206.6	4	14.2
Complement C1q subcomponent subunit C	C1QC_HUMAN	25.8	8.6	SCX, SDS, IEF	SDS	313.6	6	21.2
Complement C1q tumor necrosis factor-related protein 5	C1QT5_HUMAN	25.3	6.1	SDS, IEF	SDS	187.0	3	19.8
Complement C1s subcomponent	C1S_HUMAN	76.6	4.8	IEF		46.4	1	1.2
Complement C2	CO2_HUMAN	83.2	7.2	IEF		35.0	1	1.7
Complement C3	CO3_HUMAN	187.0	6.0	SCX, SDS, IEF	IEF	2880.5	50	35.1
Complement C4-A	CO4A_HUMAN	192.7	6.7	IEF		707.8	13	10.1
Complement C4-B	CO4B_HUMAN	192.6	6.9	SCX, SDS	SDS	277.6	6	4.6
Complement C5	CO5_HUMAN	188.2	6.1	IEF		46.9	1	0.6
Complement component 1 Q subcomponent-binding protein, mitochondrial	C1QBP_HUMAN	31.3	4.7	IEF		56.3	1	5.0
Complement component C6	CO6_HUMAN	104.7	6.4	IEF		279.6	6	7.3
Complement component C7	CO7_HUMAN	93.5	6.1	IEF		116.4	3	5.2
Complement component C8 alpha chain	CO8A_HUMAN	65.1	6.1	SDS, IEF	IEF	537.8	9	12.3
Complement component C8 beta chain	CO8B_HUMAN	67.0	8.5	SCX, SDS, IEF	IEF	421.6	8	17.1
Complement component C8 gamma chain	CO8G_HUMAN	22.3	8.5	SCX, SDS, IEF	SDS	475.3	9	50.5
Complement component C9	CO9_HUMAN	63.1	5.4	SCX, SDS, IEF	IEF	1750.2	26	37.9
Complement factor B	CFAB_HUMAN	85.5	6.7	SCX, SDS, IEF	IEF	743.1	13	22.6
Complement factor D	CFAD_HUMAN	27.0	7.6	SCX, SDS, IEF	IEF	300.7	4	31.6
Complement factor H	CFAH_HUMAN	139.0	6.2	SCX, SDS, IEF	IEF	1063.1	21	17.2
Complement factor H-related protein 1	FHR1_HUMAN	37.6	7.4	SDS, IEF	SDS	816.8	11	19.1
Complement factor H-related protein 2	FHR2_HUMAN	30.6	6.0	SDS, IEF	SDS	650.3	8	17.4
Complement factor I	CFAI_HUMAN	65.7	7.7	IEF		75.5	1	2.2
Connective tissue growth factor	CTGF_HUMAN	38.1	8.4	SDS, IEF	IEF	72.2	2	7.4
Copine-3	CPNE3_HUMAN	60.1	5.6	SDS		37.0	1	1.7
Core histone macro-H2A.1	H2AY_HUMAN	39.6	9.8	IEF		41.1	1	5.4
Core histone macro-H2A.2	H2AW_HUMAN	40.0	9.7	SDS		30.6	1	3.2
Coronin-1C	COR1C_HUMAN	53.2	6.6	SCX		51.1	1	3.4
Corticosteroid-binding globulin	CBG_HUMAN	45.1	5.6	IEF		41.5	1	4.0
Creatine kinase B-type	KCRB_HUMAN	42.6	5.3	IEF		112.1	2	6.3
Cullin-3	CUL3_HUMAN	88.9	8.7	IEF		30.8	1	2.5
Cullin-associated NEDD8-dissociated protein 1	CAND1_HUMAN	136.3	5.5	IEF		48.6	1	0.9
Cystathionine beta-synthase-like protein	CBSL_HUMAN	60.5	6.2	IEF		70.3	2	8.5
Cystatin-B	CYTB_HUMAN	11.1	7.0	SCX, SDS, IEF	IEF	104.9	2	24.5
Cystatin-C	CYTC_HUMAN	15.8	9.0	SDS, IEF	SDS	137.0	2	18.5
Cytochrome c	CYC_HUMAN	11.7	9.6	SCX, SDS	SDS	43.8	1	10.5
Cytochrome c1, heme protein, mitochondrial	CY1_HUMAN	35.4	9.2	IEF		31.7	1	4.9
Cytoplasmic dynein 1 heavy chain 1	DYHC1_HUMAN	532.1	6.0	IEF		128.2	3	0.9
Cytosol aminopeptidase	AMPL_HUMAN	56.1	8.0	SCX, IEF	IEF	191.2	4	9.6
Cytosolic non-specific dipeptidase	CNDP2_HUMAN	52.8	5.7	IEF		185.2	4	12.6
D-3-phosphoglycerate dehydrogenase	SERA_HUMAN	56.6	6.3	SDS		32.7	1	1.5
DBF4-type zinc finger-containing protein 2	ZDBF2_HUMAN	265.5	5.8	SDS		36.0	1	0.5
DDRGK domain-containing protein 1	DDRGK_HUMAN	35.6	5.1	IEF		106.6	2	9.9
Decorin	PGS2_HUMAN	39.7	8.8	SCX, SDS, IEF	IEF	362.4	6	17.8
Deleted in autism protein 1	DIA1_HUMAN	49.5	8.8	SDS, IEF	IEF	227.8	4	12.6
Delta-aminolevulinic acid dehydratase	HEM2_HUMAN	36.3	6.3	SDS, IEF	IEF	104.9	2	8.8
Dentin matrix acidic phosphoprotein 1	DMP1_HUMAN	55.7	4.0	SCX, SDS, IEF	IEF	684.7	10	10.1
Dentin sialophosphoprotein	DSPP_HUMAN	131.1	3.6	SCX, SDS, IEF	IEF	1444.9	22	6.2
Dermatopontin	DERM_HUMAN	24.0	4.7	SCX, SDS, IEF	SDS	448.8	9	45.3
Dermcidin	DCD_HUMAN	11.3	6.1	SCX, SDS, IEF	SDS	178.2	2	25.5
Dermokine	DMKN_HUMAN	47.1	6.8	SDS		50.2	1	3.6
Desmin	DESM_HUMAN	53.5	5.2	SDS		146.9	3	6.2

Desmocollin-1	DSC1_HUMAN	99.9	5.2	SDS		80.3	2	3.1
Desmocollin-3	DSC3_HUMAN	99.9	5.8	SDS		90.7	2	2.6
Desmoglein-1	DSG1_HUMAN	113.7	4.9	SDS, IEF	SDS	466.3	8	12.5
Desmoplakin	DESP_HUMAN	331.6	6.4	SDS		1610.2	32	14.4
Diablo homolog, mitochondrial	DBLOH_HUMAN	27.1	5.7	IEF		99.4	2	8.8
Dickkopf-related protein 3	DKK3_HUMAN	38.4	4.6	IEF		72.5	2	9.4
Dihydropteridine reductase	DHPR_HUMAN	25.8	6.9	SCX, IEF	IEF	64.8	2	11.5
Dihydropyrimidinase-related protein 1	DPYL1_HUMAN	62.1	6.5	SDS, IEF	IEF	112.0	3	5.8
Dihydropyrimidinase-related protein 2	DPYL2_HUMAN	62.3	6.0	SDS, IEF	IEF	299.1	6	16.3
Diphosphoinositol polyphosphate phosphohydrolase 2	NUDT4_HUMAN	20.3	6.0	IEF		31.7	1	5.6
DNA-(apurinic or apyrimidinic site) lyase	APEX1_HUMAN	35.5	8.3	IEF		39.7	1	5.3
DnaJ homolog subfamily C member 3	DNJC3_HUMAN	57.5	5.8	SCX, SDS, IEF	IEF	1036.1	14	30.4
Dolichyl-diphosphooligosaccharide--protein glycosyltransferase subunit 2	RPN2_HUMAN	69.2	5.4	IEF		41.1	1	1.9
Dynactin subunit 1	DCTN1_HUMAN	141.6	5.6	SCX, IEF	IEF	44.3	1	1.1
Dynactin subunit 2	DCTN2_HUMAN	44.2	5.1	IEF		59.2	2	5.7
Echinoderm microtubule-associated protein-like 4	EMAL4_HUMAN	108.8	6.0	IEF		32.8	1	0.9
Ectonucleotide pyrophosphatase/phosphodiesterase family member 2	ENPP2_HUMAN	98.9	7.1	SCX, IEF	IEF	135.2	4	4.3
EGF-containing fibulin-like extracellular matrix protein 1	FBLN3_HUMAN	54.6	5.0	IEF		74.6	2	6.3
EGF-containing fibulin-like extracellular matrix protein 2	FBLN4_HUMAN	49.4	4.8	SDS, IEF	SDS	90.4	2	5.4
EH domain-containing protein 2	EHD2_HUMAN	61.1	6.0	IEF		66.2	1	2.2
Elongation factor 1-alpha 1	EF1A1_HUMAN	50.1	9.1	SDS, IEF	IEF	98.5	3	8.2
Elongation factor 1-gamma	EF1G_HUMAN	50.1	6.2	IEF		52.5	1	3.0
Elongation factor 2	EF2_HUMAN	95.3	6.4	IEF		63.6	1	1.3
EMILIN-1	EMIL1_HUMAN	106.6	5.1	SCX, SDS, IEF	IEF	489.9	9	10.0
EMILIN-2	EMIL2_HUMAN	115.6	6.0	IEF		100.7	2	2.3
Enamelin	ENAM_HUMAN	128.7	6.4	IEF		37.6	1	0.7
Endoplasmic reticulum resident protein 29	ERP29_HUMAN	29.0	6.8	SCX		31.5	1	3.8
Endoplasmic reticulum resident protein 44	ERP44_HUMAN	46.9	5.1	IEF		48.4	1	2.5
Endoplasmin	ENPL_HUMAN	92.4	4.8	SCX, SDS, IEF	IEF	2562.4	43	35.0
Endosialin	CD248_HUMAN	80.8	5.2	IEF		31.9	1	1.3
Enolase-phosphatase E1	ENOPH_HUMAN	28.9	4.7	SDS, IEF	IEF	42.5	1	8.4
Epididymal secretory protein E1	NPC2_HUMAN	16.6	7.6	IEF		136.2	3	25.8
Eukaryotic initiation factor 4A-I	IF4A1_HUMAN	46.1	5.3	SDS, IEF	SDS	51.2	1	2.5
Eukaryotic translation initiation factor 2-alpha kinase 3	E2AK3_HUMAN	125.1	5.3	IEF		42.0	1	1.0
Eukaryotic translation initiation factor 3 subunit C	EIF3C_HUMAN	105.3	5.5	IEF		51.1	1	1.0
Eukaryotic translation initiation factor 3 subunit D	EIF3D_HUMAN	63.9	5.8	IEF		68.9	1	1.8
Eukaryotic translation initiation factor 4 gamma 1	IF4G1_HUMAN	175.4	5.3	SCX		31.4	1	0.6
Eukaryotic translation initiation factor 5A-1-like	IF5AL_HUMAN	16.8	4.8	IEF		35.7	1	7.8
Extended synaptotagmin-1	ESYT1_HUMAN	122.8	5.6	SCX, IEF	IEF	43.6	1	1.2
Extracellular matrix protein 1	ECM1_HUMAN	60.6	6.2	IEF		36.6	1	1.9
Extracellular matrix protein 2	ECM2_HUMAN	79.7	5.3	SCX, SDS, IEF	IEF	834.5	17	21.2
Extracellular serine/threonine protein kinase FAM20C	FA20C_HUMAN	66.2	7.7	SCX, IEF	IEF	144.1	4	7.9
Extracellular superoxide dismutase [Cu-Zn]	SODE_HUMAN	25.8	6.1	SCX, SDS, IEF	SDS	397.9	6	26.7
Ezrin	EZRI_HUMAN	69.4	5.9	SDS		180.1	4	6.1
Fatty acid-binding protein, adipocyte	FABP4_HUMAN	14.7	6.6	IEF		32.6	1	9.1
Fatty acid-binding protein, epidermal	FABP5_HUMAN	15.2	6.6	SDS		249.1	4	16.3
Ferritin heavy chain	FRIH_HUMAN	21.2	5.3	SDS, IEF	IEF	200.2	4	14.2
Ferritin light chain	FRIL_HUMAN	20.0	5.5	SCX, SDS, IEF	IEF	169.3	2	17.1
Fibrillin-1	FBN1_HUMAN	312.0	4.8	SCX, IEF	IEF	266.5	7	2.4
Fibrillin-2	FBN2_HUMAN	314.6	4.7	IEF		172.8	4	1.6
Fibrinogen alpha chain	FIBA_HUMAN	94.9	5.7	IEF		33.5	1	1.5
Fibrinogen beta chain	FIBB_HUMAN	55.9	8.5	IEF		74.4	2	4.5
Fibroblast growth factor receptor 1	FGFR1_HUMAN	91.8	5.8	SCX, SDS, IEF	IEF	718.6	11	9.6
Fibroleukin	FGL2_HUMAN	50.2	7.1	SCX, IEF	IEF	292.6	5	8.2
Fibromodulin	FMOD_HUMAN	43.2	5.7	SCX, SDS, IEF	IEF	748.6	12	28.2
Fibronectin	FINC_HUMAN	262.5	5.5	SCX, SDS, IEF	IEF	1464.7	27	13.3
Fibulin-1	FBLN1_HUMAN	77.2	5.1	IEF		112.3	2	5.0
Fibulin-2	FBLN2_HUMAN	126.5	4.7	IEF		44.0	1	1.5
Fibulin-5	FBLN5_HUMAN	50.1	4.6	IEF		37.3	1	2.0
Filaggrin-2	FILA2_HUMAN	247.9	8.4	SDS, IEF	SDS	160.4	5	4.4
Filamin-A	FLNA_HUMAN	280.6	5.7	SCX, SDS, IEF	IEF	1279.4	26	14.7
Filamin-B	FLNB_HUMAN	278.0	5.5	SCX, SDS, IEF	IEF	506.6	12	7.2
Flavin reductase (NADPH)	BLVRB_HUMAN	22.1	7.1	IEF		108.6	1	7.3
Follistatin	FST_HUMAN	38.0	5.5	IEF		44.4	1	3.8

Fructose-bisphosphate aldolase A	ALDOA_HUMAN	39.4	8.3	SDS, IEF	IEF	117.7	2	6.3
Fructose-bisphosphate aldolase C	ALDOC_HUMAN	39.4	6.4	SCX, SDS, IEF	IEF	95.1	1	4.4
Fumarate hydratase, mitochondrial	FUMH_HUMAN	54.6	8.8	IEF		35.1	1	3.5
Galectin-1	LEG1_HUMAN	14.7	5.3	SCX, SDS, IEF	IEF	251.8	5	32.6
Galectin-3	LEG3_HUMAN	26.1	8.6	SCX, SDS, IEF	IEF	151.3	3	12.8
Galectin-3-binding protein	LG3BP_HUMAN	65.3	5.1	IEF		42.0	1	2.9
Galectin-7	LEG7_HUMAN	15.1	7.0	SDS		247.1	4	39.0
Gamma-aminobutyric acid receptor-associated protein-like 2	GBRL2_HUMAN	13.7	7.8	IEF		33.0	1	10.3
Gamma-enolase	ENOG_HUMAN	47.2	4.9	IEF		339.8	5	18.2
Gamma-glutamyltransferase 5	GGT5_HUMAN	62.2	7.2	SDS, IEF	SDS	79.0	1	2.0
Gelsolin	GELS_HUMAN	85.6	5.9	SCX, SDS, IEF	IEF	843.0	13	15.1
General vesicular transport factor p115	USO1_HUMAN	107.8	4.8	IEF		32.7	1	1.2
Glia-derived nexin	GDN_HUMAN	44.0	9.3	IEF		32.5	1	2.5
Glucagon	GLUC_HUMAN	20.9	5.8	IEF		30.4	1	5.6
Glucose-6-phosphate isomerase	G6PI_HUMAN	63.1	8.4	SCX, SDS, IEF	IEF	242.2	3	8.2
Glucosidase 2 subunit beta	GLU2B_HUMAN	59.4	4.3	SCX, SDS, IEF	IEF	609.6	11	19.7
Glutaminyl-peptide cyclotransferase	QPCT_HUMAN	40.9	6.1	IEF		66.8	1	4.2
Glutamyl aminopeptidase	AMPE_HUMAN	109.2	5.3	SDS, IEF	IEF	63.8	1	1.3
Glutathione peroxidase 3	GPX3_HUMAN	25.5	8.3	IEF		131.0	3	11.5
Glutathione reductase, mitochondrial	GSHR_HUMAN	56.2	8.7	SCX, SDS, IEF	IEF	67.0	1	2.3
Glutathione S-transferase Mu 2	GSTM2_HUMAN	25.7	6.0	IEF		89.1	2	14.2
Glutathione S-transferase Mu 3	GSTM3_HUMAN	26.5	5.4	IEF		63.0	2	12.4
Glutathione S-transferase omega-1	GSTO1_HUMAN	27.5	6.2	SCX, SDS, IEF	IEF	53.1	1	4.1
Glutathione S-transferase P	GSTP1_HUMAN	23.3	5.4	SCX, SDS, IEF	SDS	413.1	7	53.8
Glyceraldehyde-3-phosphate dehydrogenase	G3P_HUMAN	36.0	8.6	SCX, SDS, IEF	IEF	1259.1	16	48.7
Glycogen phosphorylase, brain form	PYGB_HUMAN	96.6	6.4	IEF		65.2	2	2.4
Glycogen phosphorylase, liver form	PYGL_HUMAN	97.1	6.7	SDS, IEF	IEF	63.1	2	2.6
Glypican-1	GPC1_HUMAN	61.6	7.1	IEF		36.0	1	2.5
Glypican-4	GPC4_HUMAN	62.4	6.3	IEF		51.3	1	2.7
Golgi integral membrane protein 4	GOLI4_HUMAN	81.8	4.7	IEF		64.2	1	1.9
Growth arrest-specific protein 1	GAS1_HUMAN	35.7	5.3	IEF		45.0	1	3.2
Growth arrest-specific protein 6	GAS6_HUMAN	79.6	5.8	SCX, SDS, IEF	IEF	1432.7	19	28.7
Guanine nucleotide-binding protein G(i) subunit alpha-2	GNAI2_HUMAN	40.4	5.3	IEF		79.0	2	7.6
Guanine nucleotide-binding protein G(I)/G(S)/G(T) subunit beta-2	GBB2_HUMAN	37.3	5.6	SDS, IEF	IEF	40.0	1	3.2
Guanylate-binding protein 2	GBP2_HUMAN	67.2	5.5	IEF		65.0	1	2.7
Guanylate-binding protein 3	GBP3_HUMAN	68.1	6.1	SDS		30.1	1	1.8
Haptoglobin	HPT_HUMAN	45.2	6.1	SDS, IEF	IEF	420.7	9	25.6
HEAT repeat-containing protein 1	HEAT1_HUMAN	242.2	6.1	SDS, IEF	SDS	35.8	1	0.4
Heat shock 70 kDa protein 1-like	HS71L_HUMAN	70.3	5.8	SDS		174.8	3	6.4
Heat shock 70 kDa protein 1A	HS71A_HUMAN	70.0	5.5	SCX, SDS, IEF	IEF	437.8	9	19.8
Heat shock 70 kDa protein 4	HSP74_HUMAN	94.3	5.1	IEF		43.0	1	1.3
Heat shock cognate 71 kDa protein	HSP7C_HUMAN	70.9	5.4	IEF		654.8	12	21.5
Heat shock protein beta-1	HSPB1_HUMAN	22.8	6.0	SDS, IEF	SDS	145.2	2	12.7
Heat shock protein HSP 90-alpha	HS90A_HUMAN	84.6	4.9	SCX, SDS, IEF	IEF	743.5	13	18.9
Heat shock protein HSP 90-beta	HS90B_HUMAN	83.2	5.0	SCX, SDS, IEF	IEF	863.4	15	17.3
Heat shock-related 70 kDa protein 2	HSP72_HUMAN	70.0	5.6	SDS, IEF	IEF	412.3	7	11.3
Heme-binding protein 2	HEBP2_HUMAN	22.9	4.5	SDS, IEF	IEF	106.2	2	13.7
Hemoglobin subunit alpha	HBA_HUMAN	15.2	8.7	SCX, SDS, IEF	IEF	736.8	8	55.6
Hemoglobin subunit beta	HBB_HUMAN	16.0	6.7	SCX, SDS, IEF	IEF	1062.7	14	78.9
Hemoglobin subunit delta	HBD_HUMAN	16.0	7.8	SDS, IEF	IEF	533.0	8	46.9
Hemopexin	HEMO_HUMAN	51.6	6.5	SDS, IEF	IEF	746.8	12	36.1
Heparan sulfate glucosamine 3-O-sulfotransferase 3B1	HS3SB_HUMAN	43.3	9.7	IEF		69.8	1	2.8
Heparin cofactor 2	HEP2_HUMAN	57.0	6.4	SCX, SDS, IEF	IEF	376.8	8	14.4
Heterogeneous nuclear ribonucleoprotein A1-like 2	RA1L2_HUMAN	34.2	9.1	IEF		182.0	4	13.1
Heterogeneous nuclear ribonucleoprotein C-like 3	HNRC3_HUMAN	32.0	5.5	IEF		154.0	3	8.2
Heterogeneous nuclear ribonucleoprotein K	HNRPK_HUMAN	50.9	5.4	IEF		84.2	2	6.3
Heterogeneous nuclear ribonucleoprotein Q	HNRPQ_HUMAN	69.6	8.7	IEF		34.3	1	2.1
Heterogeneous nuclear ribonucleoprotein U	HNRPU_HUMAN	90.5	5.8	IEF		51.7	2	4.8
Heterogeneous nuclear ribonucleoprotein U-like protein 1	HNRL1_HUMAN	95.7	6.5	IEF		43.1	1	1.5
Heterogeneous nuclear ribonucleoproteins A2/B1	ROA2_HUMAN	37.4	9.0	IEF		163.1	3	13.3
Hippocalcin-like protein 1	HPCL1_HUMAN	22.3	5.2	IEF		46.0	1	6.2
Histidine-rich glycoprotein	HRG_HUMAN	59.5	7.1	SCX, SDS, IEF	IEF	780.1	13	19.8
Histone H1.0	H10_HUMAN	20.9	10.8	SDS		38.0	1	6.7
Histone H1.2	H12_HUMAN	21.4	10.9	SDS, IEF	IEF	137.8	3	11.3

Histone H2A type 1-H	H2A1H_HUMAN	13.9	10.9	SCX, SDS, IEF	SDS	204.8	2	21.9
Histone H2A type 2-B	H2A2B_HUMAN	14.0	10.9	SDS		94.9	2	24.6
Histone H2A.V	H2AV_HUMAN	13.5	10.6	SCX, SDS, IEF	IEF	127.2	3	25.8
Histone H2B type 1-H	H2B1H_HUMAN	13.9	10.3	SDS		551.2	8	26.2
Histone H2B type 1-J	H2B1J_HUMAN	13.9	10.3	IEF		654.6	10	27.0
Histone H2B type 1-K	H2B1K_HUMAN	13.9	10.3	SCX		247.1	3	11.9
Histone H3.3	H33_HUMAN	15.3	11.3	IEF		147.2	2	28.7
Histone H3.3C	H3C_HUMAN	15.2	11.1	SCX, SDS	SDS	50.1	1	5.2
Histone H4	H4_HUMAN	11.4	11.4	SCX, SDS, IEF	IEF	411.6	6	40.8
Hornerin	HORN_HUMAN	282.2	10.0	SDS		160.4	3	4.1
Hsc70-interacting protein	F10A1_HUMAN	41.3	5.2	IEF		160.0	4	14.1
Hyaluronan-binding protein 2	HABP2_HUMAN	62.6	6.1	SCX, SDS, IEF	IEF	361.9	8	16.3
Hypoxanthine-guanine phosphoribosyltransferase	HPRT_HUMAN	24.6	6.2	IEF		66.4	2	9.6
Ig alpha-1 chain C region	IGHA1_HUMAN	37.6	6.1	SDS, IEF	IEF	361.8	6	30.3
Ig gamma-1 chain C region	IGHG1_HUMAN	36.1	8.5	SCX, SDS, IEF	SDS	1857.1	21	55.8
Ig gamma-2 chain C region	IGHG2_HUMAN	35.9	7.7	SCX, SDS, IEF	IEF	1166.4	17	46.6
Ig gamma-3 chain C region	IGHG3_HUMAN	41.3	8.2	SCX, SDS, IEF	IEF	1161.9	16	36.6
Ig gamma-4 chain C region	IGHG4_HUMAN	35.9	7.2	SDS, IEF	IEF	1126.9	15	30.9
Ig heavy chain V-I region V35	HV103_HUMAN	13.0	9.6	IEF		31.4	1	10.3
Ig heavy chain V-II region NEWM	HV207_HUMAN	12.8	6.7	SDS		52.7	1	13.7
Ig heavy chain V-III region 23	HV303_HUMAN	12.6	8.5	SCX		50.5	1	9.4
Ig heavy chain V-III region GAL	HV320_HUMAN	12.7	8.7	SDS, IEF	SDS	31.0	1	9.5
Ig heavy chain V-III region NIE	HV309_HUMAN	13.2	9.8	SDS		98.1	2	13.4
Ig heavy chain V-III region TEI	HV316_HUMAN	12.8	8.7	SCX, SDS, IEF	IEF	66.9	1	16.0
Ig heavy chain V-III region TIL	HV304_HUMAN	12.3	9.2	SDS, IEF	IEF	74.7	1	16.5
Ig heavy chain V-III region TRO	HV301_HUMAN	13.4	9.7	SDS, IEF	IEF	45.4	1	15.6
Ig kappa chain C region	IGKC_HUMAN	11.6	5.6	SCX, SDS, IEF	SDS	1482.7	13	80.2
Ig kappa chain V-I region AG	KV101_HUMAN	12.0	5.7	SDS, IEF	SDS	384.3	4	31.5
Ig kappa chain V-I region AU	KV102_HUMAN	11.9	5.1	SDS		339.9	4	31.5
Ig kappa chain V-I region CAR	KV104_HUMAN	11.7	9.5	IEF		98.9	2	16.8
Ig kappa chain V-I region DEE	KV105_HUMAN	11.7	9.4	IEF		237.5	4	16.7
Ig kappa chain V-I region Lay	KV113_HUMAN	11.8	8.0	SCX, SDS	SCX	49.4	1	8.3
Ig kappa chain V-I region Ni	KV121_HUMAN	12.2	5.2	SDS		200.2	1	14.3
Ig kappa chain V-I region Scw	KV117_HUMAN	11.8	5.7	SDS		329.0	4	27.8
Ig kappa chain V-I region Wes	KV119_HUMAN	11.6	6.9	IEF		45.4	1	16.7
Ig kappa chain V-II region MIL	KV203_HUMAN	11.9	9.4	IEF		99.8	1	11.6
Ig kappa chain V-II region TEW	KV204_HUMAN	12.3	5.7	SDS		139.1	2	32.7
Ig kappa chain V-III region B6	KV301_HUMAN	11.6	9.3	SDS		133.0	2	16.7
Ig kappa chain V-III region VG (Fragment)	KV309_HUMAN	12.6	4.9	SDS, IEF	SDS	46.3	1	7.8
Ig kappa chain V-III region WOL	KV305_HUMAN	11.7	9.1	SDS, IEF	SDS	255.7	3	39.4
Ig kappa chain V-IV region Len	KV402_HUMAN	12.6	7.9	SDS, IEF	SDS	227.0	3	23.7
Ig lambda chain V-I region NIG-64	LV104_HUMAN	11.4	4.7	SDS		42.1	1	7.2
Ig lambda chain V-I region WAH	LV106_HUMAN	11.7	6.3	SCX, IEF	IEF	60.4	1	11.9
Ig lambda chain V-III region LOI	LV302_HUMAN	11.9	4.9	SDS, IEF	SDS	251.8	3	14.4
Ig lambda chain V-III region SH	LV301_HUMAN	11.4	6.0	IEF		49.4	1	16.7
Ig lambda chain V-IV region Hil	LV403_HUMAN	11.5	6.0	SDS		34.4	1	17.8
Ig lambda-1 chain C regions	LAC1_HUMAN	11.3	7.9	SDS		366.8	5	65.1
Ig lambda-3 chain C regions	LAC3_HUMAN	11.2	6.9	SCX, SDS, IEF	SDS	409.4	5	65.1
Ig mu heavy chain disease protein	MUCB_HUMAN	43.0	5.1	IEF		91.9	2	6.6
Importin-5	IPO5_HUMAN	123.5	4.8	IEF		64.1	2	2.6
Inhibin beta A chain	INHBA_HUMAN	47.4	8.3	IEF		71.6	1	4.2
Inositol-3-phosphate synthase 1	INO1_HUMAN	61.0	5.5	SCX		44.6	1	2.3
Insulin-like growth factor I	IGF1_HUMAN	21.8	9.8	SDS, IEF	IEF	78.7	2	7.2
Insulin-like growth factor II	IGF2_HUMAN	20.1	9.5	SDS, IEF	SDS	174.3	3	18.3
Insulin-like growth factor-binding protein 1	IBP1_HUMAN	27.9	5.1	SCX, SDS, IEF	IEF	221.0	3	8.5
Insulin-like growth factor-binding protein 3	IBP3_HUMAN	31.7	9.0	SCX, SDS, IEF	IEF	425.0	9	25.8
Insulin-like growth factor-binding protein 4	IBP4_HUMAN	27.9	6.8	IEF		50.1	1	4.7
Insulin-like growth factor-binding protein 5	IBP5_HUMAN	30.5	8.6	SCX, SDS, IEF	SDS	681.9	12	29.8
Insulin-like growth factor-binding protein 7	IBP7_HUMAN	29.1	8.2	IEF		47.6	1	4.6
Insulin-like growth factor-binding protein complex acid labile subunit	ALS_HUMAN	66.0	6.3	SCX, SDS, IEF	IEF	884.8	16	28.6
Integral membrane protein 2B	ITM2B_HUMAN	30.3	5.0	SDS, IEF	IEF	139.9	3	12.4
Integrin alpha-2	ITA2_HUMAN	129.2	5.2	IEF		67.8	1	1.1
Integrin-linked protein kinase	ILK_HUMAN	51.4	8.3	IEF		49.2	1	3.1
Inter-alpha-trypsin inhibitor heavy chain H1	ITIH1_HUMAN	101.3	6.3	SCX, IEF	IEF	300.1	5	7.2

Inter-alpha-trypsin inhibitor heavy chain H2	ITIH2_HUMAN	106.4	6.4	SCX, SDS, IEF	IEF	461.9	8	14.1
Inter-alpha-trypsin inhibitor heavy chain H4	ITIH4_HUMAN	103.3	6.5	SDS, IEF	IEF	197.7	6	7.0
Interleukin enhancer-binding factor 2	ILF2_HUMAN	43.0	5.2	SDS, IEF	IEF	67.1	2	6.2
Junction plakoglobin	PLAK_HUMAN	81.7	5.8	SDS, IEF	SDS	1133.5	18	27.8
Kallikrein-4	KLK4_HUMAN	27.0	4.8	IEF		74.8	1	4.7
Kallistatin	KAIN_HUMAN	48.5	7.3	SCX, SDS, IEF	IEF	178.7	5	13.6
Kazal-type serine protease inhibitor domain-containing protein 1	KAZD1_HUMAN	32.9	4.7	IEF		39.4	1	6.6
Keratin, type I cytoskeletal 10	K1C10_HUMAN	58.8	5.1	SCX, SDS, IEF	SDS	3411.2	39	54.1
Keratin, type I cytoskeletal 13	K1C13_HUMAN	49.6	4.9	SDS		625.7	10	11.8
Keratin, type I cytoskeletal 14	K1C14_HUMAN	51.5	5.1	SCX, SDS, IEF	SDS	2624.2	40	58.1
Keratin, type I cytoskeletal 16	K1C16_HUMAN	51.2	5.0	SCX, SDS	SDS	3213.9	40	63.8
Keratin, type I cytoskeletal 17	K1C17_HUMAN	48.1	5.0	SDS		1521.1	24	42.6
Keratin, type I cytoskeletal 9	K1C9_HUMAN	62.0	5.1	SCX, SDS, IEF	SDS	3116.9	41	69.2
Keratin, type II cytoskeletal 1	K2C1_HUMAN	66.0	8.2	SCX, SDS, IEF	SDS	3589.4	42	56.1
Keratin, type II cytoskeletal 1b	K2C1B_HUMAN	61.9	5.7	SDS, IEF	SDS	513.7	9	11.1
Keratin, type II cytoskeletal 2 epidermal	K22E_HUMAN	65.4	8.1	SCX, SDS, IEF	SDS	2449.8	33	51.5
Keratin, type II cytoskeletal 4	K2C4_HUMAN	57.2	6.3	SDS		455.5	9	17.4
Keratin, type II cytoskeletal 5	K2C5_HUMAN	62.3	7.6	SCX, SDS, IEF	SDS	2130.0	32	39.0
Keratin, type II cytoskeletal 6A	K2C6A_HUMAN	60.0	8.1	SDS		3586.0	44	54.8
Keratin, type II cytoskeletal 6B	K2C6B_HUMAN	60.0	8.1	SDS		3506.3	45	56.6
Keratin, type II cytoskeletal 6C	K2C6C_HUMAN	60.0	8.1	SDS, IEF	SDS	3734.1	46	56.6
Keratin, type II cytoskeletal 78	K2C78_HUMAN	56.8	5.8	SDS, IEF	SDS	150.8	2	4.6
Kinase D-interacting substrate of 220 kDa	KDIS_HUMAN	196.4	6.2	IEF		31.7	1	0.6
Kinase suppressor of Ras 1	KSR1_HUMAN	102.1	8.9	SCX, IEF	IEF	35.3	1	0.9
Kinectin	KTN1_HUMAN	156.2	5.5	IEF		124.7	2	2.6
Kininogen-1	KNG1_HUMAN	71.9	6.3	SCX, SDS, IEF	IEF	3734.7	40	27.3
L-lactate dehydrogenase A chain	LDHA_HUMAN	36.7	8.4	SCX, SDS, IEF	IEF	205.2	3	12.0
L-lactate dehydrogenase B chain	LDHB_HUMAN	36.6	5.7	SDS, IEF	IEF	184.0	4	17.4
Lactadherin	MFGM_HUMAN	43.1	8.5	SCX, SDS, IEF	IEF	422.9	10	28.7
Lactoylglutathione lyase	LGUL_HUMAN	20.8	5.1	IEF		37.2	1	5.4
Lamin-B2	LMNB2_HUMAN	69.9	5.5	SCX, IEF	IEF	254.3	5	9.4
Laminin subunit alpha-5	LAMA5_HUMAN	399.5	6.7	SCX		32.9	1	0.2
Laminin subunit beta-2	LAMB2_HUMAN	195.9	6.1	IEF		33.5	1	0.6
Latent-transforming growth factor beta-binding protein 3	LTBP3_HUMAN	139.3	5.7	SCX, SDS, IEF	IEF	1156.2	17	12.5
Leucine-rich alpha-2-glycoprotein	A2GL_HUMAN	38.2	6.5	IEF		168.4	3	10.4
Leucine-rich repeat-containing protein 17	LRC17_HUMAN	51.8	8.5	SCX, SDS, IEF	IEF	180.5	4	12.7
Leukocyte cell-derived chemotaxin-2	LECT2_HUMAN	16.4	9.5	SDS, IEF	SDS	68.9	2	11.3
Leukocyte elastase inhibitor	ILEU_HUMAN	42.7	5.9	SCX, SDS, IEF	SDS	232.8	5	18.2
Leukotriene A-4 hydrolase	LKHA4_HUMAN	69.2	5.8	IEF		53.0	1	2.9
Lipopolysaccharide-binding protein	LBP_HUMAN	53.3	6.2	SCX, SDS, IEF	IEF	244.6	5	10.0
Lumican	LUM_HUMAN	38.4	6.2	SCX, SDS, IEF	IEF	1983.2	29	44.1
Lupus La protein	LA_HUMAN	46.8	6.7	IEF		48.8	1	3.4
Lymphoid-restricted membrane protein	LRMP_HUMAN	62.1	5.6	SDS		34.4	1	1.4
Lysine-specific demethylase 2B	KDM2B_HUMAN	152.5	8.9	SDS, IEF	IEF	38.1	1	0.6
Lysosome-associated membrane glycoprotein 2	LAMP2_HUMAN	44.9	5.4	SCX, SDS, IEF	IEF	95.2	2	4.9
Lysozyme C	LYSC_HUMAN	16.5	9.4	SCX, SDS, IEF	SDS	1054.1	15	40.5
Lysyl oxidase homolog 4	LOXL4_HUMAN	84.4	7.3	IEF		47.4	1	1.9
Macrophage migration inhibitory factor	MIF_HUMAN	12.5	7.7	IEF		34.2	1	7.8
Macrophage-capping protein	CAPG_HUMAN	38.5	5.8	SDS, IEF	IEF	108.0	2	5.7
Malate dehydrogenase, cytoplasmic	MDHC_HUMAN	36.4	6.9	SDS, IEF	IEF	207.0	4	16.5
Malate dehydrogenase, mitochondrial	MDHM_HUMAN	35.5	8.9	SDS, IEF	IEF	118.2	3	14.5
Matrix extracellular phosphoglycoprotein	MEPE_HUMAN	58.4	8.6	IEF		41.1	1	2.1
Matrix Gla protein	MGP_HUMAN	12.3	9.7	SCX, SDS, IEF	IEF	1328.0	24	24.3
Matrix metalloproteinase-14	MMP14_HUMAN	65.9	7.6	SCX, SDS, IEF	IEF	76.7	2	5.0
Matrix metalloproteinase-20	MMP20_HUMAN	54.4	8.9	SCX, SDS, IEF	IEF	3293.6	43	45.8
Matrix-remodeling-associated protein 8	MXRA8_HUMAN	49.1	6.7	SCX		36.2	1	3.8
Melanoma inhibitory activity protein 3	MIA3_HUMAN	213.6	4.8	SCX, SDS, IEF	IEF	245.8	4	2.7
Melanoma-derived growth regulatory protein	MIA_HUMAN	14.5	9.0	IEF		35.5	1	7.6
Membrane primary amine oxidase	AOC3_HUMAN	84.6	6.0	SCX, IEF	IEF	66.1	2	2.4
Membrane-associated progesterone receptor component 1	PGRC1_HUMAN	21.7	4.5	SCX		38.0	1	7.2
Metalloproteinase inhibitor 1	TIMP1_HUMAN	23.2	8.5	SCX, SDS, IEF	SDS	1093.5	16	71.5
Metalloproteinase inhibitor 2	TIMP2_HUMAN	24.4	7.5	SCX, SDS, IEF	IEF	109.5	2	15.9
Metalloproteinase inhibitor 3	TIMP3_HUMAN	24.1	9.0	SDS, IEF	IEF	270.6	5	30.3
Microtubule-associated protein 1B	MAP1B_HUMAN	270.5	4.7	SDS, IEF	IEF	294.9	7	3.8

Microtubule-associated proteins 1A/1B light chain 3 beta 2	MP3B2_HUMAN	14.6	8.7	IEF		72.8	2	16.8
Microtubule-associated proteins 1A/1B light chain 3A	MLP3A_HUMAN	14.3	8.7	SDS		46.1	1	5.8
Midkine	MK_HUMAN	15.6	9.8	SCX, SDS, IEF	SCX	162.3	3	7.0
Mimecan	MIME_HUMAN	33.9	5.5	SCX, SDS, IEF	IEF	1253.8	17	42.3
Moesin	MOES_HUMAN	67.8	6.1	SCX, SDS, IEF	IEF	608.1	14	18.4
Monocyte differentiation antigen CD14	CD14_HUMAN	40.1	5.8	SCX, SDS, IEF	IEF	372.3	7	25.6
Multiple inositol polyphosphate phosphatase 1	MINP1_HUMAN	55.0	7.9	SDS, IEF	IEF	62.8	2	5.3
Myelin protein P0	MYP0_HUMAN	27.5	9.6	SDS, IEF	SDS	164.7	4	18.5
Myosin light chain kinase, smooth muscle	MYLK_HUMAN	210.6	5.9	IEF		31.2	1	0.6
Myosin light polypeptide 6	MYL6_HUMAN	16.9	4.6	IEF		119.3	3	29.8
Myosin regulatory light chain 12B	ML12B_HUMAN	19.8	4.7	SDS, IEF	IEF	252.6	5	34.3
Myosin-10	MYH10_HUMAN	228.9	5.4	IEF		301.5	6	4.4
Myosin-2	MYH2_HUMAN	222.9	5.6	SCX		193.9	4	2.8
Myosin-9	MYH9_HUMAN	226.4	5.5	SCX, IEF	IEF	644.3	12	8.6
Myotrophin	MTPN_HUMAN	12.9	5.3	IEF		53.2	1	14.4
N-acetyl-beta-glucosaminyl-glycoprotein 4-beta-N-acetylgalactosaminyltransferase 1	B4GN4_HUMAN	116.4	6.5	IEF		68.0	2	2.2
N-acetyl-D-glucosamine kinase	NAGK_HUMAN	37.4	5.8	IEF		47.3	1	2.0
N-acetylmuramoyl-L-alanine amidase	PGRP2_HUMAN	62.2	7.3	IEF		75.7	2	6.6
N(G),N(G)-dimethylarginine dimethylaminohydrolase 2	DDAH2_HUMAN	29.6	5.7	SDS		64.9	2	10.9
Nephronectin	NPNT_HUMAN	61.9	8.7	SCX, SDS, IEF	IEF	112.2	2	5.0
Neprilysin	NEP_HUMAN	85.5	5.5	SDS, IEF	SDS	149.6	4	8.1
Nestin	NEST_HUMAN	177.3	4.3	SCX, SDS, IEF	IEF	280.7	4	3.6
Neuroblastoma suppressor of tumorigenicity 1	NBL1_HUMAN	19.4	5.1	IEF		41.0	1	3.9
Neurofilament light polypeptide	NFL_HUMAN	61.5	4.6	IEF		111.4	2	3.5
Neurosecretory protein VGF	VGF_HUMAN	67.2	4.8	SCX, IEF	IEF	180.6	2	6.3
Neutral alpha-glucosidase AB	GANAB_HUMAN	106.8	5.7	IEF		36.3	1	1.6
Nidogen-1	NID1_HUMAN	136.3	5.1	IEF		51.8	2	1.8
Nidogen-2	NID2_HUMAN	151.2	5.1	IEF		31.1	1	0.8
Nucleobindin-1	NUCB1_HUMAN	53.8	5.1	SCX, SDS, IEF	IEF	952.7	17	35.6
Nucleobindin-2	NUCB2_HUMAN	50.2	5.0	SCX, SDS, IEF	IEF	1248.7	19	35.0
Nucleolar protein 58	NOP58_HUMAN	59.5	9.0	IEF		30.2	1	2.8
Nucleolin	NUCL_HUMAN	76.6	4.6	IEF		262.2	6	9.2
Nucleophosmin	NPM_HUMAN	32.6	4.6	IEF		119.2	2	7.1
Nucleoside diphosphate kinase A	NDKA_HUMAN	17.1	5.8	SCX, IEF	IEF	102.1	2	19.1
Nucleotide exchange factor SIL1	SIL1_HUMAN	52.1	5.3	IEF		142.1	3	7.4
Olfactomedin-like protein 1	OLFL1_HUMAN	45.9	8.3	SCX, SDS, IEF	IEF	987.6	19	30.8
Olfactomedin-like protein 3	OLFL3_HUMAN	46.0	6.2	SCX, SDS, IEF	IEF	1135.4	18	38.4
Osteocalcin	OSTCN_HUMAN	11.0	6.6	SCX, IEF	IEF	127.5	1	19.0
Osteomodulin	OMD_HUMAN	49.5	5.3	SCX, SDS, IEF	IEF	4411.6	42	24.2
Osteopontin	OSTP_HUMAN	35.4	4.4	SCX, SDS, IEF	IEF	811.8	12	25.2
Pancreatic alpha-amylase	AMYP_HUMAN	57.7	6.6	IEF		98.0	2	5.5
Parathymosin	PTMS_HUMAN	11.5	4.1	SCX		53.7	1	10.8
Pentraxin-related protein PTX3	PTX3_HUMAN	41.9	4.9	SDS, IEF	IEF	369.0	6	17.6
Peptidyl-glycine alpha-amidating monooxygenase	AMD_HUMAN	108.3	6.0	SCX, SDS, IEF	IEF	483.7	8	6.2
Peptidyl-prolyl cis-trans isomerase A	PPIA_HUMAN	18.0	7.7	SCX, SDS, IEF	IEF	276.6	5	32.1
Peptidyl-prolyl cis-trans isomerase B	PPIB_HUMAN	23.7	9.4	SCX, SDS, IEF	IEF	858.5	17	44.9
Peptidyl-prolyl cis-trans isomerase FKBP10	FKB10_HUMAN	64.2	5.4	IEF		58.2	2	4.8
Peptidyl-prolyl cis-trans isomerase FKBP7	FKBP7_HUMAN	30.0	6.1	SDS, IEF	IEF	56.5	1	5.0
Periostin	POSTN_HUMAN	93.3	7.3	SCX, SDS, IEF	IEF	2698.3	37	43.5
Peripheral plasma membrane protein CASK	CSKP_HUMAN	105.1	6.0	SCX		30.6	1	1.3
Peripherin	PERI_HUMAN	53.6	5.4	IEF		226.9	5	11.3
Peroxiredoxin-1	PRDX1_HUMAN	22.1	8.3	SCX, SDS, IEF	IEF	291.0	6	27.6
Peroxiredoxin-2	PRDX2_HUMAN	21.9	5.7	SCX, SDS, IEF	IEF	322.1	6	23.7
Peroxiredoxin-4	PRDX4_HUMAN	30.5	5.9	IEF		83.5	2	7.7
Peroxiredoxin-6	PRDX6_HUMAN	25.0	6.0	SCX, SDS, IEF	IEF	310.5	6	26.3
Phosphate-regulating neutral endopeptidase	PHEX_HUMAN	86.4	8.9	SCX, SDS, IEF	IEF	93.3	3	4.8
Phosphatidylethanolamine-binding protein 1	PEBP1_HUMAN	21.0	7.0	SDS, IEF	IEF	224.4	3	35.3
Phosphatidylethanolamine-binding protein 4	PEBP4_HUMAN	25.7	6.1	SCX, SDS, IEF	IEF	202.3	5	28.6
Phosphatidylinositol 4-kinase alpha	PI4KA_HUMAN	236.7	6.6	IEF		38.3	1	0.6
Phosphatidylinositol-binding clathrin assembly protein	PICAL_HUMAN	70.7	7.7	IEF		37.9	1	1.8
Phosphoglucomutase-2	PGM2_HUMAN	68.2	6.3	IEF		31.4	1	2.9
Phosphoglycerate kinase 1	PGK1_HUMAN	44.6	8.3	SCX, SDS, IEF	IEF	390.0	7	27.1
Phosphoglycerate mutase 1	PGAM1_HUMAN	28.8	6.7	SDS		146.9	3	22.4
Phosphoglycerate mutase 2	PGAM2_HUMAN	28.7	9.0	SCX, IEF	SCX	49.9	1	4.3

Phospholipase B-like 1	PLBL1_HUMAN	63.2	9.1	SCX, SDS, IEF	IEF	65.6	1	1.8
Pigment epithelium-derived factor	PEDF_HUMAN	46.3	6.0	SCX, SDS, IEF	IEF	4814.7	54	67.7
Plakophilin-1	PKP1_HUMAN	82.8	9.3	SDS		175.1	5	8.2
Plasma kallikrein	KLKB1_HUMAN	71.3	8.6	SDS		62.8	1	1.6
Plasma protease C1 inhibitor	IC1_HUMAN	55.1	6.1	SCX, SDS, IEF	IEF	269.9	5	12.8
Plasma serine protease inhibitor	IPSP_HUMAN	45.6	9.3	SCX, SDS, IEF	IEF	271.7	7	14.3
Plasminogen	PLMN_HUMAN	90.5	7.0	SCX, SDS, IEF	IEF	1715.1	29	40.4
Plastin-3	PLST_HUMAN	70.8	5.4	SDS, IEF	IEF	394.6	7	11.9
Platelet basic protein	CXCL7_HUMAN	13.9	9.0	IEF		81.1	2	18.8
Platelet-activating factor acetylhydrolase IB subunit beta	PA1B2_HUMAN	25.6	5.6	SCX, IEF	IEF	81.0	2	12.2
Platelet-derived growth factor D	PDGFD_HUMAN	42.8	8.3	IEF		31.0	1	3.5
Platelet-derived growth factor subunit A	PDGFA_HUMAN	24.0	9.5	SDS, IEF	IEF	42.8	1	3.8
Plectin	PLEC_HUMAN	531.5	5.7	IEF		494.1	11	3.4
Pleiotrophin	PTN_HUMAN	18.9	9.7	SCX, SDS, IEF	SDS	400.0	6	23.8
Plexin domain-containing protein 2	PXDC2_HUMAN	59.5	6.0	IEF		88.6	1	2.3
Poly [ADP-ribose] polymerase 1	PARP1_HUMAN	113.0	9.0	IEF		48.5	1	1.3
Poly(rC)-binding protein 1	PCBP1_HUMAN	37.5	6.7	IEF		38.8	1	3.7
Poly(rC)-binding protein 2	PCBP2_HUMAN	38.6	6.3	IEF		43.2	1	3.6
Polymerase I and transcript release factor	PTRF_HUMAN	43.4	5.5	IEF		143.4	2	8.5
Pre-B-cell leukemia transcription factor-interacting protein 1	PBIP1_HUMAN	80.6	5.2	SCX, SDS, IEF	IEF	288.3	5	9.0
Prelamin-A/C	LMNA_HUMAN	74.1	6.6	SCX, SDS, IEF	IEF	1015.1	18	28.9
Prenylcysteine oxidase 1	PCYOX_HUMAN	56.6	5.8	IEF		41.2	1	3.0
Probable tubulin polyglutamylase TTLL1	TTLL1_HUMAN	49.0	8.9	SDS		41.7	1	1.9
Procollagen C-endopeptidase enhancer 1	PCOC1_HUMAN	47.9	7.4	SCX, SDS, IEF	IEF	3346.6	35	61.7
Procollagen C-endopeptidase enhancer 2	PCOC2_HUMAN	45.7	8.8	SCX, SDS, IEF	IEF	101.7	2	5.3
Procollagen-lysine,2-oxoglutarate 5-dioxygenase 1	PLOD1_HUMAN	83.5	6.5	IEF		141.5	5	8.4
Profilin-1	PROF1_HUMAN	15.0	8.4	IEF		110.5	2	21.4
Programmed cell death 6-interacting protein	PDC6I_HUMAN	96.0	6.1	IEF		42.5	1	2.0
Prohibitin-2	PHB2_HUMAN	33.3	9.8	IEF		70.4	2	10.0
Prolargin	PRELP_HUMAN	43.8	9.5	SCX, SDS, IEF	IEF	905.2	16	28.0
Prolow-density lipoprotein receptor-related protein 1	LRP1_HUMAN	504.3	5.2	SCX, IEF	IEF	130.3	3	0.8
Prolyl 3-hydroxylase 2	P3H2_HUMAN	80.9	5.5	IEF		34.2	1	1.0
Prolyl 4-hydroxylase subunit alpha-1	P4HA1_HUMAN	61.0	5.7	IEF		42.6	1	1.9
Prolyl 4-hydroxylase subunit alpha-2	P4HA2_HUMAN	60.9	5.5	IEF		88.6	2	6.0
Proprotein convertase subtilisin/kexin type 5	PCSK5_HUMAN	206.8	5.7	IEF		47.6	1	0.7
Proprotein convertase subtilisin/kexin type 9	PCSK9_HUMAN	74.2	6.1	IEF		66.9	1	1.6
ProSAAS	PCSK1_HUMAN	27.4	6.2	SCX, IEF	IEF	182.1	3	12.3
Prosaposin	SAP_HUMAN	58.1	5.1	IEF		37.2	1	1.7
Proteasome activator complex subunit 1	PSME1_HUMAN	28.7	5.8	IEF		36.0	1	4.8
Proteasome subunit alpha type-2	PSA2_HUMAN	25.9	6.9	IEF		31.0	1	8.1
Proteasome subunit alpha type-5	PSA5_HUMAN	26.4	4.7	IEF		118.2	2	10.4
Proteasome subunit alpha type-6	PSA6_HUMAN	27.4	6.3	IEF		34.8	1	4.9
Proteasome subunit alpha type-7	PSA7_HUMAN	27.9	8.6	SDS, IEF	IEF	84.4	2	14.5
Proteasome subunit beta type-1	PSB1_HUMAN	26.5	8.3	SDS		70.1	2	10.4
Proteasome subunit beta type-3	PSB3_HUMAN	22.9	6.1	SCX		34.7	1	8.8
Protein	AMBP_HUMAN	75.5	4.8	IEF		65.7	2	4.9
Protein AMBP	CNPY2_HUMAN	39.0	5.9	SCX, SDS, IEF	IEF	473.5	7	22.2
Protein canopy homolog 2	CNPY4_HUMAN	20.6	4.8	SDS, IEF	IEF	160.2	4	29.7
Protein canopy homolog 4	PARK7_HUMAN	28.3	4.6	SCX, SDS, IEF	IEF	207.4	3	14.5
Protein deglycase DJ-1	PDIA3_HUMAN	19.9	6.3	IEF		111.8	3	16.4
Protein disulfide-isomerase	PDIA4_HUMAN	57.1	4.8	SCX, SDS, IEF	IEF	2213.8	32	49.6
Protein disulfide-isomerase A3	PDIA6_HUMAN	56.7	6.0	SCX, SDS, IEF	IEF	837.0	15	31.7
Protein disulfide-isomerase A4	PDIA1_HUMAN	72.9	5.0	SCX, SDS, IEF	IEF	1140.8	20	34.1
Protein disulfide-isomerase A6	F198B_HUMAN	48.1	5.0	SCX, SDS, IEF	IEF	825.2	12	30.9
Protein FAM198B	LZIC_HUMAN	57.5	9.8	IEF		187.1	5	11.2
Protein LZIC	NDRG1_HUMAN	21.5	4.9	IEF		50.0	1	4.2
Protein NDRG1	OS9_HUMAN	42.8	5.5	SCX		30.7	1	4.1
Protein phosphatase 1 regulatory subunit 42	PPR42_HUMAN	40.9	6.8	SDS		33.6	1	2.8
Protein phosphatase 1 regulatory subunit 7	PP1R7_HUMAN	41.5	4.8	SDS, IEF	SDS	81.5	2	8.1
Protein S100-A11	S10AB_HUMAN	11.7	6.6	IEF		122.5	2	25.7
Protein S100-A13	S10AD_HUMAN	11.5	5.9	SCX, IEF	IEF	151.3	2	21.4
Protein S100-A8	S10A8_HUMAN	10.8	6.5	IEF		48.4	1	11.8
Protein S100-A9	S10A9_HUMAN	13.2	5.7	SCX, IEF	IEF	138.8	2	24.6
Protein S100-B	S100B_HUMAN	10.7	4.5	SCX, IEF	IEF	97.2	2	16.3

Protein SZT2	SZT2_HUMAN	377.8	5.9	IEF		60.1	2	0.3
Protein Wnt-10a	WN10A_HUMAN	46.4	9.4	IEF		54.8	1	2.6
Protein Z-dependent protease inhibitor	ZPI_HUMAN	50.7	8.3	SCX, SDS, IEF	IEF	1896.1	29	43.2
Protein-arginine deiminase type-2	PADI2_HUMAN	75.5	5.4	SCX, IEF	IEF	41.8	1	2.6
Protein-L-isoaspartate(D-aspartate) O-methyltransferase	PIMT_HUMAN	24.6	6.7	IEF		50.8	1	4.0
Protein-lysine 6-oxidase	LYOX_HUMAN	46.9	8.4	SCX, SDS, IEF	IEF	163.4	3	10.3
Prothrombin	THRB_HUMAN	70.0	5.6	SCX, SDS, IEF	IEF	9358.8	102	62.7
Protocadherin gamma-C4	PCDGL_HUMAN	101.2	5.2	SCX		35.6	1	1.6
Putative high mobility group protein B1-like 1	HGB1A_HUMAN	24.2	5.9	IEF		42.1	1	7.1
Putative hydroxypyruvate isomerase	HYI_HUMAN	30.4	5.4	SDS, IEF	IEF	75.6	1	6.1
Putative keratin-87 protein	KR87P_HUMAN	29.1	5.6	IEF		57.8	2	4.7
Putative lipocalin 1-like protein 1	LC1L1_HUMAN	17.9	4.9	SDS, IEF	IEF	67.1	1	6.8
Putative nascent polypeptide-associated complex subunit alpha-like protein	NACP1_HUMAN	23.3	4.5	IEF		47.9	1	6.1
Putative protein FAM10A4	ST134_HUMAN	27.4	5.0	SDS		69.1	1	5.8
Pyruvate kinase PKM	KPYM_HUMAN	57.9	8.0	SDS, IEF	IEF	398.5	8	23.2
Quinone oxidoreductase	QOR_HUMAN	35.2	8.6	IEF		65.8	2	7.6
Rab GDP dissociation inhibitor alpha	GDIA_HUMAN	50.6	5.0	IEF		180.8	4	12.3
Rab GDP dissociation inhibitor beta	GDIB_HUMAN	50.6	6.1	SDS, IEF	IEF	145.4	4	13.3
Ras GTPase-activating-like protein IQGAP1	IQGA1_HUMAN	189.1	6.1	IEF		174.1	2	1.9
Ras-related protein Rab-10	RAB10_HUMAN	22.5	8.6	IEF		56.4	1	5.5
Ras-related protein Rab-2A	RAB2A_HUMAN	23.5	6.1	IEF		37.5	1	6.1
Ras-related protein Rap-1b	RAP1B_HUMAN	20.8	5.6	IEF		53.0	1	6.5
Reelin	RELN_HUMAN	388.1	5.5	IEF		104.3	3	1.2
Regulator of microtubule dynamics protein 3	RMD3_HUMAN	52.1	5.0	IEF		38.1	1	3.6
Reticulocalbin-1	RCN1_HUMAN	38.9	4.9	IEF		80.7	2	8.5
Reticulocalbin-3	RCN3_HUMAN	37.5	4.7	SDS, IEF	IEF	396.0	7	24.4
Reticulon-4	RTN4_HUMAN	129.9	4.4	IEF		37.8	1	1.1
Retina-specific copper amine oxidase	AOC2_HUMAN	83.6	6.5	SDS, IEF	IEF	136.5	3	6.5
Retinal dehydrogenase 1	AL1A1_HUMAN	54.8	6.3	IEF		68.8	2	4.4
Retinoic acid receptor responder protein 2	RARR2_HUMAN	18.6	9.3	SCX, SDS, IEF	IEF	458.8	6	31.9
Retinol-binding protein 4	RET4_HUMAN	23.0	5.8	SDS, IEF	IEF	113.6	2	14.4
Rho GDP-dissociation inhibitor 1	GDIR1_HUMAN	23.2	5.0	SDS, IEF	SDS	200.6	4	22.5
Ribonuclease 4	RNAS4_HUMAN	16.8	9.3	SCX, SDS, IEF	SDS	119.0	2	12.2
Ribonuclease inhibitor	RINI_HUMAN	49.9	4.7	IEF		41.4	1	3.3
Ribonuclease pancreatic	RNAS1_HUMAN	17.6	9.1	SDS, IEF	IEF	639.5	7	43.6
Ribonuclease T2	RNT2_HUMAN	29.5	6.7	SDS, IEF	IEF	91.4	3	10.5
Ribosome-binding protein 1	RRBP1_HUMAN	152.4	8.7	IEF		76.6	2	2.1
Ribosyldihydronicotinamide dehydrogenase [quinone]	NQO2_HUMAN	25.9	5.9	SDS		34.9	1	7.8
rRNA 2'-O-methyltransferase fibrillarin	FBRL_HUMAN	33.8	10.2	IEF		97.1	3	12.1
Ryanodine receptor 2	RYR2_HUMAN	564.2	5.7	SDS		30.8	1	0.2
S-phase kinase-associated protein 1	SKP1_HUMAN	18.6	4.4	IEF		115.0	2	25.8
Sarcoplasmic reticulum histidine-rich calcium-binding protein	SRCH_HUMAN	80.2	4.6	SCX, IEF	IEF	47.8	1	2.6
Schlafen family member 5	SLFN5_HUMAN	101.0	8.5	SCX		30.2	1	0.8
Secernin-1	SCRN1_HUMAN	46.4	4.7	SDS		35.3	1	3.9
Secreted frizzled-related protein 1	SFRP1_HUMAN	35.4	9.1	SCX, IEF	IEF	114.8	2	8.9
Secreted frizzled-related protein 2	SFRP2_HUMAN	33.5	7.4	IEF		52.5	1	5.1
Secreted frizzled-related protein 3	SFRP3_HUMAN	36.2	8.8	SDS, IEF	IEF	204.6	3	9.2
Secreted phosphoprotein 24	SPP24_HUMAN	24.3	8.6	SCX, SDS, IEF	IEF	416.3	6	29.9
Secretogranin-1	SCG1_HUMAN	78.2	5.0	IEF		158.7	3	6.2
Secretogranin-2	SCG2_HUMAN	70.9	4.7	SCX, SDS, IEF	IEF	731.1	16	22.9
Secretogranin-3	SCG3_HUMAN	53.0	4.9	SCX, IEF	IEF	282.9	6	11.5
Selenium-binding protein 1	SBP1_HUMAN	52.4	5.9	SCX, SDS	SCX	86.8	2	4.9
Semaphorin-3C	SEM3C_HUMAN	85.2	9.0	IEF		30.1	1	1.5
Semaphorin-3D	SEM3D_HUMAN	89.6	7.9	SCX, SDS, IEF	IEF	222.2	5	7.6
Semaphorin-3E	SEM3E_HUMAN	89.2	7.2	SCX, SDS, IEF	IEF	717.2	14	15.9
Septin-2	SEPT2_HUMAN	41.5	6.1	IEF		127.7	2	7.8
Serine protease HTRA1	HTRA1_HUMAN	51.3	8.1	SCX, SDS, IEF	IEF	178.1	4	7.9
Serine protease inhibitor Kazal-type 5	ISK5_HUMAN	120.6	8.5	IEF		36.1	1	0.9
Serine/threonine-protein kinase 26	STK26_HUMAN	46.5	5.2	IEF		41.7	1	2.4
Serine/threonine-protein phosphatase PP1-gamma catalytic subunit	PP1G_HUMAN	37.0	6.1	SDS, IEF	SDS	83.8	3	12.7
Serotransferrin	TRFE_HUMAN	77.0	6.8	SCX, SDS, IEF	SDS	3781.0	56	62.3
Serpin B12	SPB12_HUMAN	46.2	5.4	SDS		74.6	2	6.2
Serpin B6	SPB6_HUMAN	42.6	5.2	SCX, SDS, IEF	SDS	127.6	3	12.0
Serpin B9	SPB9_HUMAN	42.4	5.6	SDS, IEF	IEF	71.3	2	5.6

Serpin H1	SERPH_HUMAN	46.4	8.7	IEF		49.0	1	2.9
Serum albumin	ALBU_HUMAN	69.3	5.9	SCX, SDS, IEF	SDS	13303.6	153	79.1
Serum amyloid P-component	SAMP_HUMAN	25.4	6.1	SCX, SDS, IEF	IEF	400.3	6	26.5
SH3 domain-binding glutamic acid-rich-like protein	SH3L1_HUMAN	12.8	5.2	IEF		43.3	1	15.8
Signal peptide, CUB and EGF-like domain-containing protein 3	SCUB3_HUMAN	109.2	7.9	SCX, IEF	IEF	593.0	9	13.1
Skin-specific protein 32	XP32_HUMAN	26.2	8.4	SDS		38.9	1	3.2
Slit homolog 3 protein	SLIT3_HUMAN	167.6	8.0	SCX, IEF	IEF	125.8	3	2.7
Small nuclear ribonucleoprotein-associated proteins B and B'	RSMB_HUMAN	24.6	11.2	IEF		37.4	1	2.9
Sodium-driven chloride bicarbonate exchanger	S4A10_HUMAN	125.9	6.0	IEF		103.6	1	1.5
Sodium/calcium exchanger 3	NAC3_HUMAN	102.9	5.0	IEF		31.4	1	0.9
Sodium/potassium-transporting ATPase subunit beta-3	AT1B3_HUMAN	31.5	8.6	SDS, IEF	SDS	31.4	1	3.9
Solute carrier family 12 member 2	S12A2_HUMAN	131.4	6.0	IEF		37.8	1	0.7
SPARC	SPRC_HUMAN	34.6	4.7	SCX, SDS, IEF	SDS	3258.3	43	47.9
SPARC-like protein 1	SPRL1_HUMAN	75.2	4.7	SCX, SDS, IEF	IEF	300.0	5	7.8
Spectrin alpha chain, non-erythrocytic 1	SPTN1_HUMAN	284.4	5.2	SCX, SDS, IEF	IEF	580.4	9	5.0
Spectrin beta chain, erythrocytic	SPTB1_HUMAN	246.3	5.1	IEF		39.3	1	0.5
Spectrin beta chain, non-erythrocytic 1	SPTB2_HUMAN	274.4	5.4	IEF		211.4	6	3.2
Spermatogenesis-associated protein 7	SPAT7_HUMAN	67.7	5.9	SDS		60.8	2	1.7
Spliceosome RNA helicase DDX39B	DX39B_HUMAN	49.0	5.4	IEF		34.8	1	2.3
Spondin-1	SPON1_HUMAN	90.9	5.9	SCX, SDS, IEF	IEF	718.8	14	23.4
Src substrate cortactin	SRC8_HUMAN	61.5	5.2	IEF		53.3	1	2.2
Stromal cell-derived factor 1	SDF1_HUMAN	10.7	9.9	IEF		111.0	2	23.7
Sulfhydryl oxidase 1	QSOX1_HUMAN	82.5	9.1	IEF		31.8	1	2.0
Superoxide dismutase [Cu-Zn]	SODC_HUMAN	15.9	5.7	SDS, IEF	IEF	148.9	2	30.5
Sushi domain-containing protein 5	SUSD5_HUMAN	68.0	4.8	SCX, IEF	IEF	114.9	2	3.5
Sushi repeat-containing protein SRPX	SRPX_HUMAN	51.5	9.0	IEF		188.4	4	7.8
Sushi repeat-containing protein SRPX2	SRPX2_HUMAN	52.9	7.0	IEF		39.4	1	3.4
Synaptic vesicle membrane protein VAT-1 homolog	VAT1_HUMAN	41.9	5.9	IEF		63.6	2	7.4
Synaptonemal complex protein SC65	SC65_HUMAN	50.3	4.7	SCX		41.0	1	2.5
Synaptotagmin-11	SYT11_HUMAN	48.3	9.2	IEF		40.1	1	3.9
Syntaxin-3	STX3_HUMAN	33.1	5.3	IEF		38.9	1	4.8
T-complex protein 1 subunit alpha	TCPA_HUMAN	60.3	5.8	IEF		33.2	1	2.0
T-complex protein 1 subunit beta	TCPB_HUMAN	57.5	6.0	IEF		67.5	2	5.6
T-complex protein 1 subunit theta	TCPQ_HUMAN	59.6	5.4	IEF		161.6	4	8.6
T-complex protein 1 subunit zeta	TCPZ_HUMAN	58.0	6.2	IEF		66.5	2	5.8
Tachykinin-3	TKNK_HUMAN	13.4	6.7	IEF		83.3	2	18.2
Talin-1	TLN1_HUMAN	269.6	5.8	IEF		249.1	4	3.4
TATA-binding protein-associated factor 172	BTAF1_HUMAN	206.8	6.1	SDS		30.1	1	0.4
TBC1 domain family member 2B	TBD2B_HUMAN	109.8	5.8	IEF		50.2	0	0.0
Tenascin	TENA_HUMAN	240.7	4.8	SCX, SDS, IEF	IEF	2761.7	44	25.0
Terminal uridylyltransferase 7	TUT7_HUMAN	171.1	6.4	SDS		36.3	1	0.7
Testican-1	TICN1_HUMAN	49.1	5.7	SCX, SDS	SDS	131.3	3	6.2
Testican-2	TICN2_HUMAN	46.7	4.7	IEF		104.6	2	5.2
Testican-3	TICN3_HUMAN	49.4	4.8	SCX, SDS, IEF	IEF	685.6	9	21.1
Tetranectin	TETN_HUMAN	22.5	5.5	SCX, SDS, IEF	IEF	1253.0	18	50.0
Thioredoxin	TXND5_HUMAN	11.7	4.8	SDS, IEF	IEF	101.9	2	22.9
Thioredoxin domain-containing protein 5	THIO_HUMAN	47.6	5.6	IEF		88.4	1	4.6
Thioredoxin-dependent peroxide reductase, mitochondrial	PRDX3_HUMAN	27.7	7.7	IEF		59.6	2	9.0
Thioredoxin-like protein 1	TXNL1_HUMAN	32.2	4.8	SCX		32.8	1	2.4
Thrombospondin-1	TSP1_HUMAN	129.3	4.7	SCX, SDS, IEF	IEF	3337.6	46	26.4
Thrombospondin-2	TSP2_HUMAN	129.9	4.6	SCX, SDS, IEF	IEF	683.9	10	8.2
Thy-1 membrane glycoprotein	THY1_HUMAN	17.9	9.0	SCX, SDS, IEF	SCX	105.8	3	16.1
Thymidine phosphorylase	TYPH_HUMAN	49.9	5.4	IEF		46.4	1	3.1
Thyroxine-binding globulin	THBG_HUMAN	46.3	5.9	IEF		38.8	1	2.2
Transcription factor HIVEP2	ZEP2_HUMAN	268.9	6.5	SDS		55.5	0	0.0
Transcription initiation factor IIA subunit 1	TF2AA_HUMAN	41.5	4.4	IEF		62.3	1	2.9
Transforming growth factor beta-1	TGFB1_HUMAN	44.3	8.8	SCX, SDS, IEF	IEF	1744.4	25	45.6
Transforming growth factor beta-2	TGFB2_HUMAN	47.7	8.8	SDS, IEF	IEF	403.1	7	19.8
Transforming growth factor beta-3	TGFB3_HUMAN	47.3	8.3	IEF		45.4	1	3.6
Transforming growth factor-beta-induced protein ig-h3	BGH3_HUMAN	74.6	7.6	SCX, SDS, IEF	IEF	3447.8	46	56.7
Transgelin-2	TAGL2_HUMAN	22.4	8.4	SCX, SDS, IEF	IEF	174.0	5	29.1
Transitional endoplasmic reticulum ATPase	TERA_HUMAN	89.3	5.1	SDS, IEF	IEF	169.2	4	6.2
Transketolase	TKT_HUMAN	67.8	7.6	IEF		87.6	2	3.9
Translin	TSN_HUMAN	26.2	6.0	IEF		37.5	1	5.3

Transmembrane protein 198	TM198_HUMAN	39.4	10.0	SDS		39.9	1	2.5
Transportin-1	TNPO1_HUMAN	102.3	4.8	IEF		37.5	1	1.3
Transthyretin	TTHY_HUMAN	15.9	5.5	SCX, SDS, IEF	IEF	1079.2	10	68.7
Triosephosphate isomerase	TPIS_HUMAN	30.8	5.6	SCX, SDS, IEF	IEF	558.5	8	42.0
Tropomyosin alpha-1 chain	TPM1_HUMAN	32.7	4.7	SDS, IEF	SDS	436.6	7	28.5
Tropomyosin alpha-3 chain	TPM3_HUMAN	32.9	4.7	SDS, IEF	SDS	275.8	4	18.6
Tropomyosin alpha-4 chain	TPM4_HUMAN	28.5	4.7	SCX, SDS, IEF	IEF	342.3	5	21.0
Tropomyosin beta chain	TPM2_HUMAN	32.8	4.7	IEF		361.7	5	12.7
Troponin T, slow skeletal muscle	TNNT1_HUMAN	32.9	5.9	IEF		39.6	1	5.0
Trypsin-1	TRY1_HUMAN	26.5	6.1	SDS, IEF	SDS	269.9	6	24.7
Tryptophan--tRNA ligase, cytoplasmic	SYWC_HUMAN	53.1	5.8	IEF		35.3	1	3.4
Tubulin alpha-1A chain	TBA1A_HUMAN	50.1	4.9	SDS, IEF	IEF	333.8	8	24.6
Tubulin alpha-1B chain	TBA1B_HUMAN	50.1	4.9	IEF		330.7	7	21.7
Tubulin alpha-1C chain	TBA1C_HUMAN	49.9	5.0	SCX, SDS	SDS	99.6	2	5.1
Tubulin alpha-3E chain	TBA3E_HUMAN	49.8	5.0	SCX		74.8	2	6.7
Tubulin beta chain	TBB5_HUMAN	49.6	4.8	IEF		641.4	11	25.2
Tubulin beta-2A chain	TBB2A_HUMAN	49.9	4.8	IEF		518.9	10	20.4
Tubulin beta-4A chain	TBB4A_HUMAN	49.6	4.8	SCX, SDS	SDS	128.2	3	7.4
Tyrosine-protein kinase receptor UFO	UFO_HUMAN	98.3	5.3	SDS, IEF	IEF	104.8	2	2.6
Tyrosine-protein phosphatase non-receptor type 9	PTN9_HUMAN	68.0	8.2	SDS		67.5	1	1.3
Ubiquitin carboxyl-terminal hydrolase isozyme L1	UCHL1_HUMAN	24.8	5.3	IEF		33.8	1	4.5
Ubiquitin-60S ribosomal protein L40	RL40_HUMAN	14.7	9.9	SCX, IEF	IEF	115.6	2	19.5
Ubiquitin-conjugating enzyme E2 L3	UB2L3_HUMAN	17.9	8.7	IEF		56.0	1	14.3
Ubiquitin-conjugating enzyme E2 variant 1	UB2V1_HUMAN	16.5	7.7	IEF		70.7	2	10.9
Ubiquitin-like modifier-activating enzyme 1	UBA1_HUMAN	117.8	5.5	SCX, SDS, IEF	IEF	197.4	4	4.8
Ubiquitin-like protein ISG15	ISG15_HUMAN	17.9	6.8	IEF		50.4	1	7.9
UHRF1-binding protein 1	URFB1_HUMAN	159.4	5.8	IEF		56.9	1	0.9
Uncharacterized protein KIAA1958	K1958_HUMAN	79.2	6.4	SDS		33.4	1	1.0
Unconventional myosin-Vb	MYO5B_HUMAN	213.5	6.8	IEF		40.3	1	0.4
UPF0568 protein C14orf166	CN166_HUMAN	28.1	6.2	SCX, IEF	SCX	40.9	1	5.7
V-type proton ATPase catalytic subunit A	VATA_HUMAN	68.3	5.3	SCX		43.4	1	1.9
V-type proton ATPase subunit B, brain isoform	VATB2_HUMAN	56.5	5.6	IEF		42.4	1	2.9
V-type proton ATPase subunit D	VATD_HUMAN	28.2	9.4	IEF		61.9	1	4.5
Vacuolar protein sorting-associated protein 13D	VP13D_HUMAN	491.6	6.1	SDS		37.5	1	0.2
Vacuolar protein sorting-associated protein 35	VPS35_HUMAN	91.6	5.3	IEF		31.1	1	1.1
Vasorin	VASN_HUMAN	71.7	7.2	IEF		41.6	1	2.4
Ventricular zone-expressed PH domain-containing protein homolog 1	MELT_HUMAN	94.7	6.3	SCX		31.1	1	0.8
Versican core protein	CSPG2_HUMAN	372.6	4.4	SCX, SDS, IEF	IEF	934.1	12	4.7
Vimentin	VIME_HUMAN	53.6	5.1	SCX, SDS, IEF	IEF	2884.4	39	41.8
Vinculin	VINC_HUMAN	123.7	5.5	IEF		253.5	5	6.4
Vitamin D-binding protein	VTDB_HUMAN	52.9	5.4	SCX, SDS, IEF	SDS	1341.3	20	47.7
Vitamin K-dependent protein C	PROC_HUMAN	52.0	5.9	SCX, SDS, IEF	IEF	1600.4	19	37.5
Vitamin K-dependent protein S	PROS_HUMAN	75.1	5.5	SCX, SDS, IEF	IEF	1998.3	26	30.0
Vitamin K-dependent protein Z	PROZ_HUMAN	44.7	5.6	SCX, SDS, IEF	IEF	1060.9	17	30.3
Vitrin	VITRN_HUMAN	73.9	9.3	SCX, SDS, IEF	IEF	651.0	11	25.1
Vitronectin	VTNC_HUMAN	54.3	5.6	SCX, SDS, IEF	IEF	1355.0	18	42.5
Voltage-dependent calcium channel subunit alpha-2/delta-1	CA2D1_HUMAN	124.5	5.1	SDS		35.6	1	0.9
Voltage-dependent calcium channel subunit alpha-2/delta-2	CA2D2_HUMAN	129.7	5.5	SDS, IEF	SDS	82.0	2	2.9
WNT1-inducible-signaling pathway protein 2	WISP2_HUMAN	26.8	8.3	SDS, IEF	IEF	44.7	1	4.0
X-ray repair cross-complementing protein 5	XRCC5_HUMAN	82.7	5.5	IEF		74.6	2	3.7
X-ray repair cross-complementing protein 6	XRCC6_HUMAN	69.8	6.2	SDS, IEF	IEF	73.8	2	3.9
Xaa-Pro dipeptidase	PEPD_HUMAN	54.5	5.6	SDS, IEF	SDS	33.8	1	2.0
Zinc transporter ZIP14	S39AE_HUMAN	54.2	5.2	IEF		72.2	2	5.1
Zinc-alpha-2-glycoprotein	ZA2G_HUMAN	34.2	5.7	SDS, IEF	IEF	240.3	4	22.1

Supplementary Table S2 Complete list of overrepresented protein classes or subclasses in human dentin as determined by the PANTHER classification system. Subordinated ontologies are listed under their parent terms and indicated by indent/arrowhead.

Protein	UniProtKB entry	Mascot score	PANTHER protein class or subclass
**Enzyme modulator (90)**			
**► Protease inhibitor (62)**			
**► Serine protease inhibitor (30)**			
Alpha-2-HS-glycoprotein	FETUA_HUMAN	7006.6	protease inhibitor
Pigment epithelium-derived factor	PEDF_HUMAN	4814.7	serine protease inhibitor
Antithrombin-III	ANT3_HUMAN	4113.0	serine protease inhibitor
Kininogen-1	KNG1_HUMAN	3734.7	protease inhibitor
Complement C3	CO3_HUMAN	2880.5	complement component, signaling molecule, serine protease inhibitor
Alpha-2-macroglobulin	A2MG_HUMAN	2761.1	complement component, signaling molecule, serine protease inhibitor
Protein Z-dependent protease inhibitor	ZPI_HUMAN	1896.1	serine protease inhibitor
Alpha-1-antitrypsin	A1AT_HUMAN	1758.0	serine protease inhibitor
Amyloid beta A4 protein	A4_HUMAN	1378.1	protease inhibitor
Basement membrane-specific heparan sulfate proteoglycan core protein	PGBM_HUMAN	1283.0	extracellular matrix protein, protease inhibitor
Metalloproteinase inhibitor 1	TIMP1_HUMAN	1093.5	protease inhibitor
Histidine-rich glycoprotein	HRG_HUMAN	780.1	protease inhibitor
Complement C4-A	CO4A_HUMAN	707.8	complement component, signaling molecule, serine protease inhibitor
Testican-3	TICN3_HUMAN	685.6	protease inhibitor
Insulin-like growth factor-binding protein 5	IBP5_HUMAN	681.9	protease inhibitor
Alpha-1-antichymotrypsin	AACT_HUMAN	654.3	serine protease inhibitor
Glucosidase 2 subunit beta	GLU2B_HUMAN	609.6	enzyme modulator
Inter-alpha-trypsin inhibitor heavy chain H2	ITIH2_HUMAN	461.9	serine protease inhibitor
Insulin-like growth factor-binding protein 3	IBP3_HUMAN	425.0	protease inhibitor
Heparin cofactor 2	HEP2_HUMAN	376.8	serine protease inhibitor
Angiotensinogen	ANGT_HUMAN	367.8	serine protease inhibitor
Alpha-1B-glycoprotein	A1BG_HUMAN	365.4	protease inhibitor
Amyloid-like protein 2	APLP2_HUMAN	342.8	protease inhibitor
Inter-alpha-trypsin inhibitor heavy chain H1	ITIH1_HUMAN	300.1	serine protease inhibitor
Complement C4-B	CO4B_HUMAN	277.6	complement component, signaling molecule, serine protease inhibitor
Plasma serine protease inhibitor	IPSP_HUMAN	271.7	serine protease inhibitor
Metalloproteinase inhibitor 3	TIMP3_HUMAN	270.6	protease inhibitor
Plasma protease C1 inhibitor	IC1_HUMAN	269.9	serine protease inhibitor
Leukocyte elastase inhibitor	ILEU_HUMAN	232.8	serine protease inhibitor
Phosphatidylethanolamine-binding protein 1	PEBP1_HUMAN	224.4	protease inhibitor
Insulin-like growth factor-binding protein 1	IBP1_HUMAN	221.0	protease inhibitor
Secreted frizzled-related protein 3	SFRP3_HUMAN	204.6	signaling molecule, protease inhibitor
Phosphatidylethanolamine-binding protein 4	PEBP4_HUMAN	202.3	protease inhibitor
Rho GDP-dissociation inhibitor 1	GDIR1_HUMAN	200.6	signaling molecule, enzyme modulator
Inter-alpha-trypsin inhibitor heavy chain H4	ITIH4_HUMAN	197.7	serine protease inhibitor
Alpha-2-antiplasmin	A2AP_HUMAN	188.8	serine protease inhibitor
ProSAAS	PCSK1_HUMAN	182.1	protease inhibitor
Rab GDP dissociation inhibitor alpha	GDIA_HUMAN	180.8	enzyme modulator
Kallistatin	KAIN_HUMAN	178.7	serine protease inhibitor
BTB/POZ domain-containing protein KCTD12	KCD12_HUMAN	176.1	enzyme modulator
Ras GTPase-activating-like protein IQGAP1	IQGA1_HUMAN	174.1	enzyme modulator
Acidic leucine-rich nuclear phosphoprotein 32 family member A	AN32A_HUMAN	161.3	enzyme modulator
Rab GDP dissociation inhibitor beta	GDIB_HUMAN	145.4	enzyme modulator
Acyl-CoA-binding protein	ACBP_HUMAN	135.9	enzyme modulator
Testican-1	TICN1_HUMAN	131.3	protease inhibitor
Septin-2	SEPT2_HUMAN	127.7	cytoskeletal protein, enzyme modulator
Serpin B6	SPB6_HUMAN	127.6	serine protease inhibitor
Secreted frizzled-related protein 1	SFRP1_HUMAN	114.8	signaling molecule, protease inhibitor
Metalloproteinase inhibitor 2	TIMP2_HUMAN	109.5	protease inhibitor
Cystatin-B	CYTB_HUMAN	104.9	protease inhibitor
Testican-2	TICN2_HUMAN	104.6	protease inhibitor
A disintegrin and metalloproteinase with thrombospondin motifs 8	ATS8_HUMAN	99.6	extracellular matrix glycoprotein, metalloprotease, serine protease inhibitor
Elongation factor 1-alpha 1	EF1A1_HUMAN	98.5	hydrolase, enzyme modulator
Cathepsin L1	CATL1_HUMAN	85.2	protease, protease inhibitor
Heterogeneous nuclear ribonucleoprotein K	HNRPK_HUMAN	84.2	ribonucleoprotein, serine protease, enzyme modulator

Guanine nucleotide-binding protein G(i) subunit alpha-2	GNAI2_HUMAN	79.0	enzyme modulator
Serpin B12	SPB12_HUMAN	74.6	serine protease inhibitor
Serpin B9	SPB9_HUMAN	71.3	serine protease inhibitor
EH domain-containing protein 2	EHD2_HUMAN	66.2	calcium-binding protein, enzyme modulator
Cathepsin O	CATO_HUMAN	65.3	protease, protease inhibitor
Guanylate-binding protein 2	GBP2_HUMAN	65.0	enzyme modulator
Importin-5	IPO5_HUMAN	64.1	enzyme modulator
Elongation factor 2	EF2_HUMAN	63.6	hydrolase, enzyme modulator
Antileukoproteinase	SLPI_HUMAN	60.5	serine protease inhibitor
Acidic leucine-rich nuclear phosphoprotein 32 family member B	AN32B_HUMAN	59.7	enzyme modulator
26S proteasome non-ATPase regulatory subunit 3	PSMD3_HUMAN	54.2	enzyme modulator
Ras-related protein Rap-1b	RAP1B_HUMAN	53.0	enzyme modulator
Secreted frizzled-related protein 2	SFRP2_HUMAN	52.5	signaling molecule, protease inhibitor
TBC1 domain family member 2B	TBD2B_HUMAN	50.2	protease, enzyme modulator
Insulin-like growth factor-binding protein 4	IBP4_HUMAN	50.1	protease inhibitor
Serpin H1	SERPH_HUMAN	49.0	serine protease inhibitor
Complement C5	CO5_HUMAN	46.9	complement component, signaling molecule, serine protease inhibitor
Follistatin	FST_HUMAN	44.4	protease inhibitor
ADAMTS-like protein 2	ATL2_HUMAN	44.2	extracellular matrix glycoprotein, metalloprotease, serine protease inhibitor
Poly(rC)-binding protein 2	PCBP2_HUMAN	43.2	ribonucleoprotein, serine protease, enzyme modulator
Agrin	AGRIN_HUMAN	42.9	extracellular matrix protein, protease inhibitor
Corticosteroid-binding globulin	CBG_HUMAN	41.5	serine protease inhibitor
Unconventional myosin-Vb	MYO5B_HUMAN	40.3	actin family cytoskeletal protein, actin binding motor protein, enzyme modulator
Guanine nucleotide-binding protein G(I)/G(S)/G(T) subunit beta-2	GBB2_HUMAN	40.0	hydrolase, enzyme modulator
Caveolin-1	CAV1_HUMAN	39.7	enzyme modulator
Poly(rC)-binding protein 1	PCBP1_HUMAN	38.8	protease, ribonucleoprotein, serine protease, enzyme modulator
Thyroxine-binding globulin	THBG_HUMAN	38.8	serine protease inhibitor
Serine protease inhibitor Kazal-type 5	ISK5_HUMAN	36.1	protease inhibitor
26S proteasome non-ATPase regulatory subunit 12	PSD12_HUMAN	35.5	enzyme modulator
1-phosphatidylinositol 4,5-bisphosphate phosphodiesterase delta-1	PLCD1_HUMAN	34.4	signaling molecule, enzyme modulator
Laminin subunit beta-2	LAMB2_HUMAN	33.5	extracellular matrix protein, protease inhibitor
Laminin subunit alpha-5	LAMA5_HUMAN	32.9	extracellular matrix protein, protease inhibitor
Glia-derived nexin	GDN_HUMAN	32.5	serine protease inhibitor
CD276 antigen	CD276_HUMAN	31.4	protease inhibitor
Guanylate-binding protein 3	GBP3_HUMAN	30.1	enzyme modulator
**Hydrolase (89) ► Protease (66) ► Metalloprotease (23) ► Serine protease (24)**			
Prothrombin	THRB_HUMAN	9358.8	serine protease
Serotransferrin	TRFE_HUMAN	3781.0	serine protease
Matrix metalloproteinase-20	MMP20_HUMAN	3293.6	metalloprotease
Coagulation factor IX	FA9_HUMAN	2407.8	serine protease
Plasminogen	PLMN_HUMAN	1715.1	serine protease
Coagulation factor X	FA10_HUMAN	1680.3	serine protease
Vitamin K-dependent protein C	PROC_HUMAN	1600.4	serine protease
Vitamin K-dependent protein Z	PROZ_HUMAN	1060.9	serine protease
Coagulation factor VII	FA7_HUMAN	1034.0	serine protease
Hemopexin	HEMO_HUMAN	746.8	metalloprotease
72 kDa type IV collagenase	MMP2_HUMAN	552.2	metalloprotease
Lactadherin	MFGM_HUMAN	422.9	cell adhesion molecule, signaling molecule, hydrolase
Haptoglobin	HPT_HUMAN	420.7	serine protease
Adipocyte enhancer-binding protein 1	AEBP1_HUMAN	408.7	metalloprotease
Hyaluronan-binding protein 2	HABP2_HUMAN	361.9	serine protease
Complement factor D	CFAD_HUMAN	300.7	extracellular matrix protein, serine protease
Dihydropyrimidinase-related protein 2	DPYL2_HUMAN	299.1	metalloprotease
Trypsin-1	TRY1_HUMAN	269.9	serine protease
Cytosol aminopeptidase	AMPL_HUMAN	191.2	metalloprotease
CD5 antigen-like	CD5L_HUMAN	188.8	oxidoreductase, serine protease
Cytosolic non-specific dipeptidase	CNDP2_HUMAN	185.2	metalloprotease
Serine protease HTRA1	HTRA1_HUMAN	178.1	chaperone, serine protease
Alkaline phosphatase, tissue-nonspecific isozyme	PPBT_HUMAN	176.3	hydrolase
Neprilysin	NEP_HUMAN	149.6	metalloprotease
ATP synthase subunit beta, mitochondrial	ATPB_HUMAN	145.7	anion channel, protease
Ectonucleotide pyrophosphatase/phosphodiesterase family member 2	ENPP2_HUMAN	135.2	hydrolase
6-phosphogluconolactonase	6PGL_HUMAN	130.7	hydrolase
Cytoplasmic dynein 1 heavy chain 1	DYHC1_HUMAN	128.2	cytoskeletal protein, hydrolase

Acid ceramidase	ASAH1_HUMAN	119.3	protease
Proteasome subunit alpha type-5	PSA5_HUMAN	118.2	protease
Dihydropyrimidinase-related protein 1	DPYL1_HUMAN	112.0	metalloprotease
Protein deglycase DJ-1	PARK7_HUMAN	111.8	transcription factor, protease
Delta-aminolevulinic acid dehydratase	HEM2_HUMAN	104.9	metalloprotease
Coagulation factor XII	FA12_HUMAN	104.2	serine protease
A disintegrin and metalloproteinase with thrombospondin motifs 8	ATS8_HUMAN	99.6	extracellular matrix glycoprotein, metalloprotease, serine protease inhibitor
Elongation factor 1-alpha 1	EF1A1_HUMAN	98.5	hydrolase, enzyme modulator
Phosphate-regulating neutral endopeptidase	PHEX_HUMAN	93.3	metalloprotease
Arginase-1	ARGI1_HUMAN	92.2	hydrolase
Cathepsin L1	CATL1_HUMAN	85.2	protease, protease inhibitor
Proteasome subunit alpha type-7	PSA7_HUMAN	84.4	protease
Heterogeneous nuclear ribonucleoprotein K	HNRPK_HUMAN	84.2	ribonucleoprotein, serine protease, enzyme modulator
Serine/threonine-protein phosphatase PP1-gamma catalytic subunit	PP1G_HUMAN	83.8	calcium-binding protein, hydrolase
5'-nucleotidase	5NTD_HUMAN	81.3	hydrolase
Gamma-glutamyltransferase 5	GGT5_HUMAN	79.0	protease
Carboxypeptidase B2	CBPB2_HUMAN	78.9	metalloprotease
Matrix metalloproteinase-14	MMP14_HUMAN	76.7	metalloprotease
Kallikrein-4	KLK4_HUMAN	74.8	serine protease
Cystathionine beta-synthase-like protein	CBSL_HUMAN	70.3	hydrolase
Proteasome subunit beta type-1	PSB1_HUMAN	70.1	protease
40S ribosomal protein S3a	RS3A_HUMAN	68.4	protease, ribosomal protein
Tyrosine-protein phosphatase non-receptor type 9	PTN9_HUMAN	67.5	hydrolase
Proprotein convertase subtilisin/kexin type 9	PCSK9_HUMAN	66.9	serine protease
Glutaminyl-peptide cyclotransferase	QPCT_HUMAN	66.8	metalloprotease
Phospholipase B-like 1	PLBL1_HUMAN	65.6	protease
Cathepsin O	CATO_HUMAN	65.3	protease, protease inhibitor
Carboxypeptidase E	CBPE_HUMAN	65.1	metalloprotease
N(G),N(G)-dimethylarginine dimethylaminohydrolase 2	DDAH2_HUMAN	64.9	hydrolase
Glutamyl aminopeptidase	AMPE_HUMAN	63.8	metalloprotease
Elongation factor 2	EF2_HUMAN	63.6	hydrolase, enzyme modulator
Plasma kallikrein	KLKB1_HUMAN	62.8	serine protease
Adenosylhomocysteinase 2	SAHH2_HUMAN	52.0	hydrolase
Alpha-L-iduronidase	IDUA_HUMAN	51.0	hydrolase
TBC1 domain family member 2B	TBD2B_HUMAN	50.2	protease, enzyme modulator
Proprotein convertase subtilisin/kexin type 5	PCSK5_HUMAN	47.6	serine protease
Complement C1s subcomponent	C1S_HUMAN	46.4	serine protease
ADAMTS-like protein 2	ATL2_HUMAN	44.2	extracellular matrix glycoprotein, metalloprotease, serine protease inhibitor
Calpain-2 catalytic subunit	CAN2_HUMAN	43.4	annexin, calmodulin, protease
V-type proton ATPase catalytic subunit A	VATA_HUMAN	43.4	anion channel, protease
Poly(rC)-binding protein 2	PCBP2_HUMAN	43.2	ribonucleoprotein, serine protease, enzyme modulator
Enolase-phosphatase E1	ENOPH_HUMAN	42.5	hydrolase
V-type proton ATPase subunit B, brain isoform	VATB2_HUMAN	42.4	anion channel, hydrolase
Guanine nucleotide-binding protein G(I)/G(S)/G(T) subunit beta-2	GBB2_HUMAN	40.0	hydrolase, enzyme modulator
Poly(rC)-binding protein 1	PCBP1_HUMAN	38.8	ribonucleoprotein, serine protease, enzyme modulator
ATP synthase subunit alpha, mitochondrial	ATPA_HUMAN	38.6	anion channel, hydrolase
Carboxypeptidase Q	CBPQ_HUMAN	38.5	metalloprotease
ATPase family AAA domain-containing protein 3C	ATD3C_HUMAN	36.3	hydrolase
Neutral alpha-glucosidase AB	GANAB_HUMAN	36.3	hydrolase
Carboxypeptidase Z	CBPZ_HUMAN	35.6	metalloprotease
Adenosylhomocysteinase	SAHH_HUMAN	35.4	hydrolase
Secernin-1	SCRN1_HUMAN	35.3	protease
Proteasome subunit alpha type-6	PSA6_HUMAN	34.8	protease
Proteasome subunit beta type-3	PSB3_HUMAN	34.7	protease
Xaa-Pro dipeptidase	PEPD_HUMAN	33.8	transcription factor, metalloprotease
Ubiquitin carboxyl-terminal hydrolase isozyme L1	UCHL1_HUMAN	33.8	protease
Diphosphoinositol polyphosphate phosphohydrolase 2	NUDT4_HUMAN	31.7	hydrolase
Aminopeptidase N	AMPN_HUMAN	31.0	metalloprotease
Proteasome subunit alpha type-2	PSA2_HUMAN	31.0	protease
Protein NDRG1	NDRG1_HUMAN	30.7	serine protease
Collagenase 3	MMP13_HUMAN	30.2	metalloprotease

Signaling molecule (54)
► Growth factor (12)

Transforming growth factor-beta-induced protein ig-h3	BGH3_HUMAN	3447.8	cell adhesion molecule, signaling molecule
SPARC	SPRC_HUMAN	3258.3	cell adhesion molecule, extracellular matrix glycoprotein, growth factor

Complement C3	CO3_HUMAN	2880.5	complement component, signaling molecule, serine protease inhibitor
Alpha-2-macroglobulin	A2MG_HUMAN	2761.1	complement component, signaling molecule, serine protease inhibitor
Periostin	POSTN_HUMAN	2698.3	cell adhesion molecule, signaling molecule
Transforming growth factor beta-1	TGFB1_HUMAN	1744.4	growth factor
Fibronectin	FINC_HUMAN	1464.7	signaling molecule
Latent-transforming growth factor beta-binding protein 3	LTBP3_HUMAN	1156.2	annexin, calmodulin, cell adhesion molecule, extracellular matrix glycoprotein, extracellular matrix structural protein, signaling molecule
Semaphorin-3E	SEM3E_HUMAN	717.2	signaling molecule
Complement C4-A	CO4A_HUMAN	707.8	complement component, signaling molecule, serine protease inhibitor
Lactadherin	MFGM_HUMAN	422.9	cell adhesion molecule, signaling molecule, hydrolase
Transforming growth factor beta-2	TGFB2_HUMAN	403.1	growth factor
Pleiotrophin	PTN_HUMAN	400.0	signaling molecule
SPARC-like protein 1	SPRL1_HUMAN	300.0	cell adhesion molecule, extracellular matrix glycoprotein, growth factor
Fibroleukin	FGL2_HUMAN	292.6	signaling molecule
Complement C4-B	CO4B_HUMAN	277.6	complement component, signaling molecule, serine protease inhibitor
Fibrillin-1	FBN1_HUMAN	266.5	annexin, calmodulin, cell adhesion molecule, extracellular matrix glycoprotein, extracellular matrix structural protein, signaling molecule
Galectin-1	LEG1_HUMAN	251.8	cell adhesion molecule, signaling molecule
Galectin-7	LEG7_HUMAN	247.1	cell adhesion molecule, signaling molecule
Melanoma inhibitory activity protein 3	MIA3_HUMAN	245.8	growth factor
Semaphorin-3D	SEM3D_HUMAN	222.2	signaling molecule
Secreted frizzled-related protein 3	SFRP3_HUMAN	204.6	signaling molecule, protease inhibitor
Rho GDP-dissociation inhibitor 1	GDIR1_HUMAN	200.6	signaling molecule, enzyme modulator
Fibrillin-2	FBN2_HUMAN	172.8	annexin, calmodulin, cell adhesion molecule, extracellular matrix glycoprotein, extracellular matrix structural protein, signaling molecule
Midkine	MK_HUMAN	162.3	signaling molecule
Galectin-3	LEG3_HUMAN	151.3	cell adhesion molecule, signaling molecule
Protein S100-A13	S10AD_HUMAN	151.3	calmodulin, signaling molecule
Protein S100-A9	S10A9_HUMAN	138.8	calmodulin, signaling molecule
Protein S100-A11	S10AB_HUMAN	122.5	calmodulin, signaling molecule
Secreted frizzled-related protein 1	SFRP1_HUMAN	114.8	signaling molecule, protease inhibitor
C-C motif chemokine 14	CCL14_HUMAN	102.1	signaling molecule
Protein S100-B	S100B_HUMAN	97.2	calmodulin, signaling molecule
EGF-containing fibulin-like extracellular matrix protein 2	FBLN4_HUMAN	90.4	annexin, calmodulin, cell adhesion molecule, extracellular matrix glycoprotein, extracellular matrix structural protein, signaling molecule
Platelet basic protein	CXCL7_HUMAN	81.1	signaling molecule
Bone morphogenetic protein 8B	BMP8B_HUMAN	77.8	growth factor
EGF-containing fibulin-like extracellular matrix protein 1	FBLN3_HUMAN	74.6	annexin, cell adhesion molecule, extracellular matrix glycoprotein, extracellular matrix structural protein, signaling molecule
Fibrinogen beta chain	FIBB_HUMAN	74.4	signaling molecule
Connective tissue growth factor	CTGF_HUMAN	72.2	growth factor
Inhibin beta A chain	INHBA_HUMAN	71.6	growth factor
Leukocyte cell-derived chemotaxin-2	LECT2_HUMAN	68.9	signaling molecule
Protein Wnt-10a	WN10A_HUMAN	54.8	signaling molecule
Secreted frizzled-related protein 2	SFRP2_HUMAN	52.5	signaling molecule, protease inhibitor
Protein S100-A8	S10A8_HUMAN	48.4	calmodulin, signaling molecule
Complement C5	CO5_HUMAN	46.9	complement component, signaling molecule, serine protease inhibitor
Transforming growth factor beta-3	TGFB3_HUMAN	45.4	growth factor
WNT1-inducible-signaling pathway protein 2	WISP2_HUMAN	44.7	growth factor
Fibulin-2	FBLN2_HUMAN	44.0	annexin, cell adhesion molecule, extracellular matrix glycoprotein, extracellular matrix structural protein, signaling molecule
Platelet-derived growth factor subunit A	PDGFA_HUMAN	42.8	growth factor
Putative high mobility group protein B1-like 1	HGB1A_HUMAN	42.1	signaling molecule, transcription factor
Membrane-associated progesterone receptor component 1	PGRC1_HUMAN	38.0	signaling molecule
Fibulin-5	FBLN5_HUMAN	37.3	annexin, calmodulin, cell adhesion molecule, extracellular matrix glycoprotein, extracellular matrix structural protein, signaling molecule
1-phosphatidylinositol 4,5-bisphosphate phosphodiesterase delta-1	PLCD1_HUMAN	34.4	signaling molecule, enzyme modulator
Platelet-derived growth factor D	PDGFD_HUMAN	31.0	growth factor
Semaphorin-3C	SEM3C_HUMAN	30.1	signaling molecule

Cytoskeletal protein (49)
- **► Actin family cytoskeletal protein (28)**
  - **► Actin binding motor protein (6)**
  - **► Actin and actin related protein (5)**
- **► Tubulin (7)**

Actin, cytoplasmic 1	ACTB_HUMAN	1952.1	actin and actin related protein
Desmoplakin	DESP_HUMAN	1610.2	cytoskeletal protein
Actin, alpha cardiac muscle 1	ACTC_HUMAN	1203.9	actin and actin related protein
Gelsolin	GELS_HUMAN	843.0	actin family cytoskeletal protein
Tubulin beta chain	TBB5_HUMAN	641.4	tubulin
Moesin	MOES_HUMAN	608.1	actin family cytoskeletal protein
Tubulin beta-2A chain	TBB2A_HUMAN	518.9	tubulin
Plectin	PLEC_HUMAN	494.1	cytoskeletal protein

Tropomyosin alpha-1 chain	TPM1_HUMAN	436.6	actin binding motor protein
Plastin-3	PLST_HUMAN	394.6	actin family cytoskeletal protein
Tropomyosin beta chain	TPM2_HUMAN	361.7	actin binding motor protein
Tropomyosin alpha-4 chain	TPM4_HUMAN	342.3	actin binding motor protein
Tubulin alpha-1A chain	TBA1A_HUMAN	333.8	tubulin
Actin, gamma-enteric smooth muscle	ACTH_HUMAN	332.3	actin and actin related protein
Tubulin alpha-1B chain	TBA1B_HUMAN	330.7	tubulin
Microtubule-associated protein 1B	MAP1B_HUMAN	294.9	cytoskeletal protein
Tropomyosin alpha-3 chain	TPM3_HUMAN	275.8	actin binding motor protein
Myosin regulatory light chain 12B	ML12B_HUMAN	252.6	actin family cytoskeletal protein, calmodulin
Adseverin	ADSV_HUMAN	211.4	actin family cytoskeletal protein
Cofilin-1	COF1_HUMAN	209.8	actin family cytoskeletal protein
Adenylyl cyclase-associated protein 1	CAP1_HUMAN	201.2	actin family cytoskeletal protein
Ezrin	EZRI_HUMAN	180.1	actin family cytoskeletal protein
Plakophilin-1	PKP1_HUMAN	175.1	cytoskeletal protein
Transgelin-2	TAGL2_HUMAN	174.0	actin family cytoskeletal protein
Filaggrin-2	FILA2_HUMAN	160.4	cytoskeletal protein
Hornerin	HORN_HUMAN	160.4	cytoskeletal protein
Cytoplasmic dynein 1 heavy chain 1	DYHC1_HUMAN	128.2	cytoskeletal protein, hydrolase
Tubulin beta-4A chain	TBB4A_HUMAN	128.2	tubulin
Septin-2	SEPT2_HUMAN	127.7	cytoskeletal protein, enzyme modulator
Actin-related protein 2	ARP2_HUMAN	123.0	actin and actin related protein
Myosin light polypeptide 6	MYL6_HUMAN	119.3	actin family cytoskeletal protein, calmodulin
Actin-related protein 2/3 complex subunit 4	ARPC4_HUMAN	110.0	actin family cytoskeletal protein
Macrophage-capping protein	CAPG_HUMAN	108.0	actin family cytoskeletal protein
Tubulin alpha-1C chain	TBA1C_HUMAN	99.6	tubulin
Tubulin alpha-3E chain	TBA3E_HUMAN	74.8	tubulin
Microtubule-associated proteins 1A/1B light chain 3 beta 2	MP3B2_HUMAN	72.8	cytoskeletal protein
Dynactin subunit 2	DCTN2_HUMAN	59.2	cytoskeletal protein
Src substrate cortactin	SRC8_HUMAN	53.3	actin family cytoskeletal protein, transcription factor
Coronin-1C	COR1C_HUMAN	51.1	actin family cytoskeletal protein
Actin-related protein 2/3 complex subunit 3	ARPC3_HUMAN	50.3	actin family cytoskeletal protein
Microtubule-associated proteins 1A/1B light chain 3A	MLP3A_HUMAN	46.1	cytoskeletal protein
Dynactin subunit 1	DCTN1_HUMAN	44.3	cytoskeletal protein
Probable tubulin polyglutamylase TTLL1	TTLL1_HUMAN	41.7	cytoskeletal protein
Actin-related protein 2/3 complex subunit 5-like protein	ARP5L_HUMAN	41.6	actin family cytoskeletal protein
Unconventional myosin-Vb	MYO5B_HUMAN	40.3	actin binding motor protein, enzyme modulator
Troponin T, slow skeletal muscle	TNNT1_HUMAN	39.6	actin binding motor protein
Actin-related protein 3C	ARP3C_HUMAN	38.3	actin and actin related protein
Gamma-aminobutyric acid receptor-associated protein-like 2	GBRL2_HUMAN	33.0	cytoskeletal protein
Actin-related protein 2/3 complex subunit 5	ARPC5_HUMAN	32.6	actin family cytoskeletal protein
**Extracellular matrix protein (33) ► Extracellular matrix glycoprotein (17) ► Extracellular matrix structural protein (10)**			
SPARC	SPRC_HUMAN	3258.3	cell adhesion molecule, extracellular matrix glycoprotein, growth factor
Chondroadherin	CHAD_HUMAN	1927.0	extracellular matrix protein
Growth arrest-specific protein 6	GAS6_HUMAN	1432.7	extracellular matrix protein
Basement membrane-specific heparan sulfate proteoglycan core protein	PGBM_HUMAN	1283.0	extracellular matrix protein, protease inhibitor
Tetranectin	TETN_HUMAN	1253.0	extracellular matrix structural protein
Latent-transforming growth factor beta-binding protein 3	LTBP3_HUMAN	1156.2	annexin, calmodulin, cell adhesion molecule, extracellular matrix glycoprotein, extracellular matrix structural protein, signaling molecule
C-type lectin domain family 11 member A	CLC11_HUMAN	1010.2	extracellular matrix structural protein
Versican core protein	CSPG2_HUMAN	934.1	extracellular matrix glycoprotein
Insulin-like growth factor-binding protein complex acid labile subunit	ALS_HUMAN	884.8	extracellular matrix protein
Spondin-1	SPON1_HUMAN	718.8	extracellular matrix glycoprotein
Signal peptide, CUB and EGF-like domain-containing protein 3	SCUB3_HUMAN	593.0	extracellular matrix glycoprotein
EMILIN-1	EMIL1_HUMAN	489.9	extracellular matrix glycoprotein
Dermatopontin	DERM_HUMAN	448.8	extracellular matrix protein
SPARC-like protein 1	SPRL1_HUMAN	300.0	cell adhesion molecule, extracellular matrix glycoprotein, growth factor
Fibrillin-1	FBN1_HUMAN	266.5	annexin, calmodulin, cell adhesion molecule, extracellular matrix glycoprotein, extracellular matrix structural protein, signaling molecule
Leucine-rich repeat-containing protein 17	LRC17_HUMAN	180.5	extracellular matrix protein
Fibrillin-2	FBN2_HUMAN	172.8	annexin, calmodulin, cell adhesion molecule, extracellular matrix glycoprotein, extracellular matrix structural protein, signaling molecule
Cartilage-associated protein	CRTAP_HUMAN	148.8	extracellular matrix protein
EMILIN-2	EMIL2_HUMAN	100.7	extracellular matrix glycoprotein
A disintegrin and metalloproteinase with thrombospondin motifs 8	ATS8_HUMAN	99.6	extracellular matrix glycoprotein, metalloprotease, serine protease inhibitor
EGF-containing fibulin-like extracellular matrix protein 2	FBLN4_HUMAN	90.4	annexin, calmodulin, cell adhesion molecule, extracellular matrix glycoprotein, extracellular matrix structural protein, signaling molecule
EGF-containing fibulin-like extracellular matrix protein 1	FBLN3_HUMAN	74.6	annexin, cell adhesion molecule, extracellular matrix glycoprotein, extracellular matrix structural protein, signaling molecule

ADAMTS-like protein 2	ATL2_HUMAN	44.2	extracellular matrix glycoprotein, metalloprotease, serine protease inhibitor
Fibulin-2	FBLN2_HUMAN	44.0	annexin, cell adhesion molecule, extracellular matrix glycoprotein, extracellular matrix structural protein, signaling molecule
Agrin	AGRIN_HUMAN	42.9	extracellular matrix protein, protease inhibitor
Bone sialoprotein 2	SIAL_HUMAN	42.8	cell adhesion molecule, extracellular matrix structural protein
Vasorin	VASN_HUMAN	41.6	extracellular matrix protein
Ameloblastin	AMBN_HUMAN	41.0	extracellular matrix protein
Synaptonemal complex protein SC65	SC65_HUMAN	41.0	extracellular matrix protein
Fibulin-5	FBLN5_HUMAN	37.3	annexin, calmodulin, cell adhesion molecule, extracellular matrix glycoprotein, extracellular matrix structural protein, signaling molecule
Prolyl 3-hydroxylase 2	P3H2_HUMAN	34.2	extracellular matrix glycoprotein
Laminin subunit beta-2	LAMB2_HUMAN	33.5	extracellular matrix protein, protease inhibitor
Laminin subunit alpha-5	LAMA5_HUMAN	32.9	extracellular matrix protein, protease inhibitor

Calcium-binding protein (28)
► Calmodulin (20)
► Annexin (11)

Nucleobindin-2	NUCB2_HUMAN	1248.7	annexin, calmodulin
Latent-transforming growth factor beta-binding protein 3	LTBP3_HUMAN	1156.2	annexin, calmodulin, cell adhesion molecule, extracellular matrix glycoprotein, extracellular matrix structural protein, signaling molecule
Calreticulin	CALR_HUMAN	1154.5	calcium-binding protein
Nucleobindin-1	NUCB1_HUMAN	952.7	annexin, calmodulin
Reticulocalbin-3	RCN3_HUMAN	396.0	calmodulin
Fibrillin-1	FBN1_HUMAN	266.5	annexin, calmodulin, cell adhesion molecule, extracellular matrix glycoprotein, extracellular matrix structural protein, signaling molecule
Myosin regulatory light chain 12B	ML12B_HUMAN	252.6	actin family cytoskeletal protein, calmodulin
Calmodulin	CALM_HUMAN	252.1	calmodulin
Calnexin	CALX_HUMAN	213.0	calcium-binding protein, chaperone
Fibrillin-2	FBN2_HUMAN	172.8	annexin, calmodulin, cell adhesion molecule, extracellular matrix glycoprotein, extracellular matrix structural protein, signaling molecule
Protein S100-A13	S10AD_HUMAN	151.3	calmodulin, signaling molecule
Protein S100-A9	S10A9_HUMAN	138.8	calmodulin, signaling molecule
Protein S100-A11	S10AB_HUMAN	122.5	calmodulin, signaling molecule
Myosin light polypeptide 6	MYL6_HUMAN	119.3	actin family cytoskeletal protein, calmodulin
Protein S100-B	S100B_HUMAN	97.2	calmodulin, signaling molecule
EGF-containing fibulin-like extracellular matrix protein 2	FBLN4_HUMAN	90.4	annexin, calmodulin, cell adhesion molecule, extracellular matrix glycoprotein, extracellular matrix structural protein, signaling molecule
Serine/threonine-protein phosphatase PP1-gamma catalytic subunit	PP1G_HUMAN	83.8	calcium-binding protein, hydrolase
Reticulocalbin-1	RCN1_HUMAN	80.7	calmodulin
EGF-containing fibulin-like extracellular matrix protein 1	FBLN3_HUMAN	74.6	annexin, cell adhesion molecule, extracellular matrix glycoprotein, extracellular matrix structural protein, signaling molecule
EH domain-containing protein 2	EHD2_HUMAN	66.2	calcium-binding protein, enzyme modulator
Calmodulin-like protein 3	CALL3_HUMAN	53.9	calmodulin
Protein S100-A8	S10A8_HUMAN	48.4	calmodulin, signaling molecule
Fibulin-2	FBLN2_HUMAN	44.0	annexin, cell adhesion molecule, extracellular matrix glycoprotein, extracellular matrix structural protein, signaling molecule
Calpain-2 catalytic subunit	CAN2_HUMAN	43.4	annexin, calmodulin, protease
Calcium-binding protein 39-like	CB39L_HUMAN	37.6	annexin
Fibulin-5	FBLN5_HUMAN	37.3	annexin, calmodulin, cell adhesion molecule, extracellular matrix glycoprotein, extracellular matrix structural protein, signaling molecule
Nidogen-2	NID2_HUMAN	31.1	calcium-binding protein
Calmodulin-like protein 5	CALL5_HUMAN	31.0	calmodulin

Oxidoreductase (28) ► Peroxidase (6)

Glyceraldehyde-3-phosphate dehydrogenase	G3P_HUMAN	1259.1	oxidoreductase
Ceruloplasmin	CERU_HUMAN	588.0	oxidoreductase
Extracellular superoxide dismutase [Cu-Zn]	SODE_HUMAN	397.9	oxidoreductase
Peroxiredoxin-2	PRDX2_HUMAN	322.1	peroxidase
Peroxiredoxin-6	PRDX6_HUMAN	310.5	peroxidase
Peroxiredoxin-1	PRDX1_HUMAN	291.0	peroxidase
Collagen triple helix repeat-containing protein 1	CTHR1_HUMAN	212.6	oxidoreductase
Malate dehydrogenase, cytoplasmic	MDHC_HUMAN	207.0	oxidoreductase
L-lactate dehydrogenase A chain	LDHA_HUMAN	205.2	oxidoreductase
CD5 antigen-like	CD5L_HUMAN	188.8	oxidoreductase, serine protease
L-lactate dehydrogenase B chain	LDHB_HUMAN	184.0	oxidoreductase
Superoxide dismutase [Cu-Zn]	SODC_HUMAN	148.9	oxidoreductase
Procollagen-lysine,2-oxoglutarate 5-dioxygenase 1	PLOD1_HUMAN	141.5	oxidoreductase
Glutathione peroxidase 3	GPX3_HUMAN	131.0	peroxidase
Malate dehydrogenase, mitochondrial	MDHM_HUMAN	118.2	oxidoreductase
Flavin reductase (NADPH)	BLVRB_HUMAN	108.6	oxidoreductase
Transketolase	TKT_HUMAN	87.6	oxidoreductase
Alcohol dehydrogenase [NADP(+)]	AK1A1_HUMAN	87.1	oxidoreductase
Peroxiredoxin-4	PRDX4_HUMAN	83.5	peroxidase
Retinal dehydrogenase 1	AL1A1_HUMAN	68.8	oxidoreductase
Glutathione reductase, mitochondrial	GSHR_HUMAN	67.0	oxidoreductase

Dihydropteridine reductase	DHPR_HUMAN	64.8	oxidoreductase
Thioredoxin-dependent peroxide reductase, mitochondrial	PRDX3_HUMAN	59.6	peroxidase
Alcohol dehydrogenase class-3	ADHX_HUMAN	46.7	oxidoreductase
Aldehyde dehydrogenase family 1 member A3	AL1A3_HUMAN	37.4	oxidoreductase
Aldose reductase	ALDR_HUMAN	37.3	oxidoreductase
D-3-phosphoglycerate dehydrogenase	SERA_HUMAN	32.7	oxidoreductase
Sulfhydryl oxidase 1	QSOX1_HUMAN	31.8	oxidoreductase
**Cell adhesion molecule (20)**			
Transforming growth factor-beta-induced protein ig-h3	BGH3_HUMAN	3447.8	cell adhesion molecule, signaling molecule
SPARC	SPRC_HUMAN	3258.3	cell adhesion molecule, extracellular matrix glycoprotein, growth factor
Periostin	POSTN_HUMAN	2698.3	cell adhesion molecule, signaling molecule
Latent-transforming growth factor beta-binding protein 3	LTBP3_HUMAN	1156.2	annexin, calmodulin, cell adhesion molecule, extracellular matrix glycoprotein, extracellular matrix structural protein, signaling molecule
Desmoglein-1	DSG1_HUMAN	466.3	cell adhesion molecule
Lactadherin	MFGM_HUMAN	422.9	cell adhesion molecule, signaling molecule, hydrolase
SPARC-like protein 1	SPRL1_HUMAN	300.0	cell adhesion molecule, extracellular matrix glycoprotein, growth factor
Fibrillin-1	FBN1_HUMAN	266.5	annexin, calmodulin, cell adhesion molecule, extracellular matrix glycoprotein, extracellular matrix structural protein, signaling molecule
Galectin-1	LEG1_HUMAN	251.8	cell adhesion molecule, signaling molecule
Galectin-7	LEG7_HUMAN	247.1	cell adhesion molecule, signaling molecule
Fibrillin-2	FBN2_HUMAN	172.8	annexin, calmodulin, cell adhesion molecule, extracellular matrix glycoprotein, extracellular matrix structural protein, signaling molecule
Myelin protein P0	MYP0_HUMAN	164.7	cell adhesion molecule
Galectin-3	LEG3_HUMAN	151.3	cell adhesion molecule, signaling molecule
Desmocollin-3	DSC3_HUMAN	90.7	cell adhesion molecule
EGF-containing fibulin-like extracellular matrix protein 2	FBLN4_HUMAN	90.4	annexin, calmodulin, cell adhesion molecule, extracellular matrix glycoprotein, extracellular matrix structural protein, signaling molecule
Desmocollin-1	DSC1_HUMAN	80.3	cell adhesion molecule
EGF-containing fibulin-like extracellular matrix protein 1	FBLN3_HUMAN	74.6	annexin, cell adhesion molecule, extracellular matrix glycoprotein, extracellular matrix structural protein, signaling molecule
Fibulin-2	FBLN2_HUMAN	44.0	annexin, cell adhesion molecule, extracellular matrix glycoprotein, extracellular matrix structural protein, signaling molecule
Bone sialoprotein 2	SIAL_HUMAN	42.8	cell adhesion molecule, extracellular matrix structural protein
Fibulin-5	FBLN5_HUMAN	37.3	annexin, calmodulin, cell adhesion molecule, extracellular matrix glycoprotein, extracellular matrix structural protein, signaling molecule
**Chaperone (17)**<br>**► Chaperonin (5)**<br>**► Hsp90 family chaperone (3)**			
Endoplasmin	ENPL_HUMAN	2562.4	Hsp90 family chaperone
Heat shock protein HSP 90-beta	HS90B_HUMAN	863.4	Hsp90 family chaperone
Heat shock protein HSP 90-alpha	HS90A_HUMAN	743.5	Hsp90 family chaperone
14-3-3 protein epsilon	1433E_HUMAN	442.5	chaperone
14-3-3 protein zeta/delta	1433Z_HUMAN	425.6	chaperone
14-3-3 protein theta	1433T_HUMAN	291.8	chaperone
Calnexin OS=Homo sapiens	CALX_HUMAN	213.0	calcium-binding protein, chaperone
14-3-3 protein beta/alpha	1433B_HUMAN	208.2	chaperone
10 kDa heat shock protein, mitochondrial	CH10_HUMAN	187.0	chaperonin
Serine protease HTRA1	HTRA1_HUMAN	178.1	chaperone, serine protease
14-3-3 protein gamma	1433G_HUMAN	174.2	chaperone
T-complex protein 1 subunit theta	TCPQ_HUMAN	161.6	chaperonin
14-3-3 protein sigma	1433S_HUMAN	152.0	chaperone
Nucleophosmin	NPM_HUMAN	119.2	chaperone
T-complex protein 1 subunit beta	TCPB_HUMAN	67.5	chaperonin
T-complex protein 1 subunit zeta	TCPZ_HUMAN	66.5	chaperonin
T-complex protein 1 subunit alpha	TCPA_HUMAN	33.2	chaperonin
**Ribosomal protein (14)**			
60S ribosomal protein L6	RL6_HUMAN	169.2	ribosomal protein
Ubiquitin-60S ribosomal protein L40	RL40_HUMAN	115.6	ribosomal protein
40S ribosomal protein S3a	RS3A_HUMAN	68.4	protease, ribosomal protein
40S ribosomal protein S24	RS24_HUMAN	56.4	ribosomal protein
40S ribosomal protein S20	RS20_HUMAN	50.5	ribosomal protein
60S ribosomal protein L27	RL27_HUMAN	48.4	ribosomal protein
60S ribosomal protein L15	RL15_HUMAN	47.9	ribosomal protein
40S ribosomal protein S7	RS7_HUMAN	46.9	ribosomal protein
40S ribosomal protein S3	RS3_HUMAN	46.5	ribosomal protein
60S ribosomal protein L14	RL14_HUMAN	43.8	ribosomal protein
60S ribosomal protein L12	RL12_HUMAN	39.5	ribosomal protein
60S acidic ribosomal protein P1	RLA1_HUMAN	38.9	ribosomal protein
Heterogeneous nuclear ribonucleoprotein Q	HNRPQ_HUMAN	34.3	ribosomal protein
60S ribosomal protein L5	RL5_HUMAN	34.3	ribosomal protein
**Histone (11)**			

Histone H2B type 1-J	H2B1J_HUMAN	654.6	histone
Histone H2B type 1-H	H2B1H_HUMAN	551.2	histone
Histone H4	H4_HUMAN	411.6	histone
Histone H2B type 1-K	H2B1K_HUMAN	247.1	histone
Histone H2A type 1-H	H2A1H_HUMAN	204.8	histone
Histone H3.3	H33_HUMAN	147.2	histone
Histone H1.2	H12_HUMAN	137.8	histone
Histone H2A type 2-B	H2A2B_HUMAN	94.9	histone
Histone H3.3C	H3C_HUMAN	50.1	histone
Core histone macro-H2A.1	H2AY_HUMAN	41.1	histone
Core histone macro-H2A.2	H2AW_HUMAN	30.6	histone
**Transcription factor (9)**			
Polymerase I and transcript release factor	PTRF_HUMAN	143.4	transcription factor
Protein deglycase DJ-1	PARK7_HUMAN	111.8	transcription factor, protease
Transcription initiation factor IIA subunit 1	TF2AA_HUMAN	62.3	transcription factor
Src substrate cortactin	SRC8_HUMAN	53.3	actin family cytoskeletal protein, transcription factor
Cullin-associated NEDD8-dissociated protein 1	CAND1_HUMAN	48.6	transcription factor
Putative nascent polypeptide-associated complex subunit alpha-like protein	NACP1_HUMAN	47.9	transcription factor
Putative high mobility group protein B1-like 1	HGB1A_HUMAN	42.1	signaling molecule, transcription factor
Ribonuclease inhibitor	RINI_HUMAN	41.4	transcription factor
Xaa-Pro dipeptidase	PEPD_HUMAN	33.8	transcription factor, metalloprotease
**Ribonucleoprotein (7)**			
rRNA 2'-O-methyltransferase fibrillarin	FBRL_HUMAN	97.1	ribonucleoprotein
Heterogeneous nuclear ribonucleoprotein K	HNRPK_HUMAN	84.2	ribonucleoprotein, serine protease, enzyme modulator
Lupus La protein	LA_HUMAN	48.8	ribonucleoprotein
Poly(rC)-binding protein 2	PCBP2_HUMAN	43.2	ribonucleoprotein, serine protease, enzyme modulator
Poly(rC)-binding protein 1	PCBP1_HUMAN	38.8	protease, ribonucleoprotein, serine protease, enzyme modulator
HEAT repeat-containing protein 1	HEAT1_HUMAN	35.8	ribonucleoprotein
Nucleolar protein 58	NOP58_HUMAN	30.2	ribonucleoprotein
**Complement component (5)**			
Complement C3	CO3_HUMAN	2880.5	complement component, signaling molecule, serine protease inhibitor
Alpha-2-macroglobulin	A2MG_HUMAN	2761.1	complement component, signaling molecule, serine protease inhibitor
Complement C4-A	CO4A_HUMAN	707.8	complement component, signaling molecule, serine protease inhibitor
Complement C4-B	CO4B_HUMAN	277.6	complement component, signaling molecule, serine protease inhibitor
Complement C5	CO5_HUMAN	46.9	complement component, signaling molecule, serine protease inhibitor
**Anion channel (4)**			
ATP synthase subunit beta, mitochondrial	ATPB_HUMAN	145.7	anion channel, protease
V-type proton ATPase catalytic subunit A	VATA_HUMAN	43.4	anion channel, protease
V-type proton ATPase subunit B, brain isoform	VATB2_HUMAN	42.4	anion channel, hydrolase
ATP synthase subunit alpha, mitochondrial	ATPA_HUMAN	38.6	anion channel, hydrolase
**Protein kinase (2)**<br>**► Non-receptor serine/threonine protein kinase (1)**			
Eukaryotic translation initiation factor 2-alpha kinase 3	E2AK3_HUMAN	42.0	non-receptor serine/threonine protein kinase
Peripheral plasma membrane protein CASK	CSKP_HUMAN	30.6	protein kinase
